# A 2 × 2 factorial, randomised, open-label trial to determine the clinical and cost-effectiveness of hypertonic saline (HTS 6%) and carbocisteine for airway clearance versus usual care over 52 weeks in adults with bronchiectasis: a protocol for the CLEAR clinical trial

**DOI:** 10.1186/s13063-019-3766-9

**Published:** 2019-12-19

**Authors:** Judy Martina Bradley, Rohan Anand, Brenda O’Neill, Kathryn Ferguson, Mike Clarke, Mary Carroll, James Chalmers, Anthony De Soyza, Jamie Duckers, Adam T. Hill, Michael R. Loebinger, Fiona Copeland, Evie Gardner, Christina Campbell, Ashley Agus, Alistair McGuire, Roisin Boyle, Fionnuala McKinney, Naomi Dickson, Danny F. McAuley, Stuart Elborn, On behalf of the CLEAR study group, Damian Downey, Martin Kelly, John Hurst, Muhammad Anwar, Rory Convery, Timothy Gatheral, Stephen Scott, Anita Sullivan, William Flight, Alina Ionescu

**Affiliations:** 1 Wellcome-Wolfson Institute For Experimental Medicine, School of Medicine, Dentistry and Biomedical Sciences, Queen’s University Belfast, Belfast, UK; 2 Centre for Health and Rehabilitation Technologies (CHaRT), Institute of Nursing and Health Research, Ulster University, Newtownabbey, UK; 3 Northern Ireland Clinical Research Network, Belfast Health and Social Care Trust, Belfast, UK; 4 Northern Ireland Methodology Hub, Centre for Public Health, School of Medicine, Dentistry and Biomedical Sciences, Queen’s University Belfast, Belfast, UK; 5 Southampton University Hospitals NHS Trust, Southampton, UK; 6 School of Medicine, University of Dundee, Dundee, UK; 7 NIHR Biomedical research centre (BRC) for Aging, Institute of Cellular Medicine, Newcastle University, Newcastle, UK; 8 Cardiff and Vale University Health Board, University Hospital Llandough, Penarth, UK; 9 Centre for Inflammation Research, University of Edinburgh, Edinburgh, UK; 10 Faculty of Medicine, National Heart and Lung Institute, Imperial College and Royal Brompton Hospital, London, UK; 11 PCD Family Support Group, Ciliopathy Alliance, London, UK; 12 Northern Ireland Clinical Trials Unit, Belfast Health and Social Care Trust, Belfast, UK; 13 Department of Health Policy, London School of Economics and Political Science, London, UK

**Keywords:** Clinical trial protocol, Factorial design, Bronchiectasis, Hypertonic saline, Carbocisteine, Exacerbation, SWAT

## Abstract

**Background:**

Current guidelines for the management of bronchiectasis (BE) highlight the lack of evidence to recommend mucoactive agents, such as hypertonic saline (HTS) and carbocisteine, to aid sputum removal as part of standard care. We hypothesise that mucoactive agents (HTS or carbocisteine, or a combination) are effective in reducing exacerbations over a 52-week period, compared to usual care.

**Methods:**

This is a 52-week, 2 × 2 factorial, randomized, open-label trial to determine the clinical effectiveness and cost effectiveness of HTS 6% and carbocisteine for airway clearance versus usual care - the Clinical and cost-effectiveness of hypertonic saline (HTS 6%) and carbocisteine for airway clearance versus usual care (CLEAR) trial. Patients will be randomised to (1) standard care and twice-daily nebulised HTS (6%), (2) standard care and carbocisteine (750 mg three times per day until visit 3, reducing to 750 mg twice per day), (3) standard care and combination of twice-daily nebulised HTS and carbocisteine, or (4) standard care. The primary outcome is the mean number of exacerbations over 52 weeks. Key inclusion criteria are as follows: adults with a diagnosis of BE on computed tomography, BE as the primary respiratory diagnosis, and two or more pulmonary exacerbations in the last year requiring antibiotics and production of daily sputum.

**Discussion:**

This trial’s pragmatic research design avoids the significant costs associated with double-blind trials whilst optimising rigour in other areas of trial delivery. The CLEAR trial will provide evidence as to whether HTS, carbocisteine or both are effective and cost effective for patients with BE.

**Trial registration:**

EudraCT number: 2017-000664-14 (first entered in the database on 20 October 2017).

ISRCTN.com, ISRCTN89040295. Registered on 6 July/2018.

Funder: National Institute for Health Research, Health Technology Assessment Programme (15/100/01).

Sponsor: Belfast Health and Social Care Trust.

Ethics Reference Number: 17/NE/0339.

Protocol version: v3.0 Final_14052018.

## Background

### Background information

Bronchiectasis (BE) is a debilitating chronic illness caused by irreversible dilatation, thickening and sac-like formations in bronchial walls. Patients usually suffer from a persistent cough, chronic daily sputum expectoration, recurrent chest infections and poor health-related quality of life (HRQoL) [1, 2]. Current estimates suggest around 5 in 1000 people in the UK have BE [3, 4] with higher numbers of patients being diagnosed with BE due to increased use of high-resolution computed tomography (HRCT) [5]. Mortality in the 52 weeks after a BE-related exacerbation is as high as 30% [6]. Morbidity is also high and UK hospitals admission data indicate that BE was the primary diagnosis in 1 in 1800 admissions, with a sevenfold increase in hospital bed days needed for treating BE in the first decade of the 21st century [7, 8].

Mucus hypersecretion is a clinical feature of BE. Airway mucosal infection often gives rise to inflammatory mediators [9], including neutrophil-derived DNA and filamentous actin, in addition to apoptotic cells and cellular debris that may collectively increase mucus production and viscosity. This mucus retention aids bacterial infection that can lead to pulmonary exacerbations, which further develops the “viscous cycle” of mucus retention, infection, inflammation and tissue damage [10]. Mucoactive drugs target this cycle by potentially increasing the ability to expectorate sputum and/or decrease mucus hypersecretion. Mucoactive drugs are classified in terms of their proposed primary mechanism of action: expectorants induce mucus expulsion, mucoregulators reduce mucus secretion, mucolytics decrease viscosity and mucokinetics increase cilia activity.

### Rationale for the study

The current guidelines indicate that mucoactive drugs plus airway clearance may be considered to enhance sputum expectoration in BE [11, 12] but Cochrane reviews have shown that the evidence to support their use is limited. Recent reviews have demonstrated that DNase and mannitol do not reduce exacerbations [13, 14]. In clinical trials, DNase increased exacerbations and resulted in a significant decrease in lung function [15]. And, as shown in a recent overview of reviews, evidence for the effectiveness of hypertonic saline (HTS) and carbocisteine is insufficient to recommend them within the management of BE [16]. However, European Multicentre Bronchiectasis Audit and Research Collaboration (EMBARC)/UK Bronchiectasis Registry (BRONCH-UK) data show that BE centres do prescribe mucoactive drugs. This is important because adherence to therapy in BE in general is low and decreases as the number of prescribed medications increases, and is also related to poorer patient outcomes, including the number of pulmonary exacerbations and quality of life [17]. Therefore, it is essential that only those drugs that are effective should be prescribed for patients with BE. There are cost considerations associated with mucoactive drugs, and there is a risk of polypharmacy side effects.

### Rationale for the interventions (use of HTS and carbocisteine)

The physiological rationale for the use of HTS in BE is based on its osmotic effects on the airway surface layer that improves airway hydration and accelerates mucus transportability, especially when combined with airway clearance techniques (ACT). There have been multiple, crossover studies exploring the use of HTS in BE [18–21] that support these effects. The only long-term (1 year) randomised, parallel-group study did not demonstrate long-term efficacy of HTS (6%) compared to placebo [22], but this “no effect” result may have been due to poor study design or lack of power, the way in which exacerbation data were collected, a true lack of effect of HTS on mucus clearance or due to chance. Thus, the need to explore the use of HTS in BE remains, and patients and practitioners need to have these important uncertainties resolved. The physiological rationale for carbocisteine is based on its ability to reduce the concentration of mucus glycoprotein, which reduces the viscosity of mucus and facilitates expectoration. As with HTS, the evidence base for carbocisteine in BE is poor. This contrasts to other respiratory conditions, where there is relatively strong evidence favouring both HTS and carbocisteine [22–24]. Therefore, the CLEAR trial will answer important clinical questions about whether similar benefits can be demonstrated in BE by using a pragmatic design to explore the specific effects of mucoactive agents, and directly support, or refute, more targeted use of these drugs.

### Rationale for comparator

In the CLEAR trial, the comparator will be standard care. In the UK, standard care is defined by the British Thoracic Society (BTS) guidelines for BE [25]. All sites will be encouraged to follow these guidelines such that all patients in the control group (as well as in the three intervention groups) will be expected to have been prescribed and taught ACT. If patients are not familiar with ACT, they will be taught the active cycle of breathing techniques [26].

### Objectives

The primary objective of the CLEAR trial is to determine whether HTS (6%) and/or carbocisteine reduces the mean number of exacerbations over 52 weeks. Secondary objectives are to determine whether HTS and/or carbocisteine improve disease-specific HRQoL, reduce time to next exacerbation, reduce number of days of antibiotics for treatment of exacerbations, improve generic HRQoL, are acceptable from a patient satisfaction perspective, are associated with adverse events (AEs) and improve lung function over 52 weeks. The study will also assess cost effectiveness and treatment adherence.

### Study design

CLEAR is a multicentre, superiority, 2 × 2 factorial, randomized, open-label trial in BE with a 52-week follow-up period. Patients will be randomised (1:1:1:1 ratio) to one of four groups: HTS alone, carbocisteine alone, HTS and carbocisteine or standard care. Additionally, all patients will be followed up at 104 weeks to explore their mucoactive drug use in the 52 weeks following the completion of the main trial. For the SPIRIT checklist, please see Additional file 8.

### Embedded sub-studies

There are three embedded sub-studies within the CLEAR trial. The first aims to validate and measure the sensitivity of the EMBARC definition of exacerbations in BE (see Additional file 1). The second sub-study will examine the use of a new home spirometer *my*SpiroSense for remote monitoring (see Additional file 3). The third will use Studies Within A Trial (SWAT) [27] to explore the effect of methods used to optimise recruitment and retention (see Additional file 4). These sub-studies will be reported separately from the main report for CLEAR.

## Methods/design

### Study setting

The CLEAR sites will include at least 16 National Health Service (NHS) hospitals in the UK with access to patients with BE managed according to BTS guidelines. Sites will include those that are part of the BRONCH-UK/or EMBARC research network [28], and additional sites will be chosen from the Northern Ireland Clinical research Network (NICRN)/National Institute for Health Research Clinical Research Network (NIHR CRN) portfolio if required. The current list of study sites is in Additional file 5.

### Internal pilot study

The main trial will be preceded by an 8-month internal pilot study in 10 sites, which will follow the processes described for the main trial with target recruitment of 60 patients. This internal pilot will be used to confirm recruitment rates, protocol compliance and data collection methods.

### Characteristics of participants

Patients will be eligible to participate in the CLEAR trial if they fulfil the following inclusion criteria: diagnosis of BE on computerised tomography (CT)/HRCT, BE as the primary respiratory diagnosis, two or more pulmonary exacerbations in the last year requiring antibiotics (including patient-reported exacerbations), production of daily sputum, stable for 14 or more days before first study visit with no changes to treatment, willing to continue any other existing medication for chronic illness throughout the study, and female participants must be either surgically sterile, postmenopausal or agree to use effective contraception during the treatment period of the trial.

The exclusion criteria are as follows: < 18 years old, cystic fibrosis (CF), chronic obstructive pulmonary disease (COPD), current smokers, female ex-smokers with > 20 pack years and male ex-smokers with > 25 pack years, forced expiratory volume in 1 s (FEV_1_) < 30%, if being treated with long term macrolides on treatment for < 1 month before joining study, regular isotonic saline, HTS, carbocisteine or any mucoactive drugs taken within the past 30 days, known intolerance or contraindication to HTS or carbocisteine, contraindications to or special warnings against the use of carbocisteine (active peptic ulceration, hereditary galactose intolerance, Lapp-lactase deficiency, glucose-galactose malabsorption), unable to swallow oral capsules, women who are pregnant or lactating, or participation in another clinical trial of an investigational medicinal product (IMP) within 30 days. Patients currently using mucoactive drugs can be considered for the CLEAR trial if they stop these for at least 30 days before being assessed for eligibility.

### Screening and informed consent

Written informed consent will be obtained by the site principal investigator or appropriately trained designee. All interested individuals who are eligible using the screening criteria will be given a participant information sheet and allowed as much time as necessary to consider the study. Informed consent will be obtained using standard procedures (Additional files 6 and 7).

### Intervention and comparator

The interventions and comparators are as follows:

- Intervention 1: standard care and twice-daily nebulised HTS (MucoClear 6%, PARI Pharma GmbH). Participants will be instructed to administer a 1 × 4 mL ampoule twice daily for 52 weeks using the eFlow rapid nebuliser and eTrack controller (PARI Pharma GmbH).
- Intervention 2: standard care and carbocisteine (750 mg three times per day until visit 3*, reducing to 750 mg two times per day) over 52 weeks.
- Intervention 3: standard care and combination of twice-daily nebulised HTS (MucoClear 6%, PARI Pharma GmbH). Participants will be instructed to administer a 1 × 4 mL ampoule twice daily for 52 weeks using the eFlow rapid nebuliser eFlow rapid nebuliser and eTrack controller (PARI Pharma GmbH). They will also be given carbocisteine (750 mg three times per day until visit 3*, reducing to 750 mg twice per day) over 52 weeks.
- Comparator: standard care over 52 weeks. Patients in the standard care group will use airway clearance techniques in the management of their BE.

*Visit 3 occurs 8 weeks (±7 days) after the baseline assessment).

### Concomitant care

Sites in this study all follow BTS guidelines for management of BE. Any prescribed medication deemed necessary to provide adequate medical care to the patient is permitted, other than as stated in the study exclusion criteria. The use of mucoactive drugs/isotonic saline outside the allocated treatment is not permitted except for short periods during exacerbations.

### Treatment discontinuation

All patients allocated to a treatment group including HTS 6% will complete a drug response assessment prior to commencing HTS in accordance with a study-specific guideline and if they do not pass this they will not continue on the study. Participants may withdraw from treatment at any time, without providing an explanation, or if discontinuation is considered by the medical team to be in the best interests of the patient. Anticipated reasons for withdrawal include intercurrent significant illness, occurrence of intolerable side effects, patient request, protocol violations or decision that the study drug should be discontinued on safety grounds. A participant may be withdrawn from the study at the discretion of the Investigator due to safety concerns.

### Study drug accountability, compliance and adherence

Patients will be asked to return any unused HTS 6% ampoules or carbocisteine at each study visit, to facilitate drug accountability. Adherence to HTS will be monitored utilising the eFlow nebuliser system with eTrack controller (which records data on nebuliser usage including frequency of use, dosage and maintenance). For the two HTS groups (intervention groups), study visit data from the eFlow nebuliser system with eTrack controller will be transferred to a Qualcomm Life 2Net Hub and subsequently to a secure Cloud-based platform. These data will not be reviewed and analysed until the end of the study, but will be checked weekly by a person not involved in study delivery, to ensure that the eFlow nebuliser system with eTrack controller is being used correctly and data are being transferred correctly.

### Outcomes

The primary outcome measure is the mean number of exacerbations over 52 weeks after randomisation. Secondary outcome measures are disease-specific HRQoL (respiratory symptoms of the domain of Quality of Life - Bronchiectasis (QoL-B) [29]), time to next exacerbation, number of days of antibiotics related to exacerbations, generic HRQoL (Euroqol 5 dimensions 5 levels (EQ-5D-5 L) [30]), measurement of health impairment using the St Georges Respiratory Questionnaire (SGRQ) [2]), health service use, quality-adjusted life years (QALYs), patient preferences for treatment, adverse events, lung function and adherence to trial treatment over 52 weeks.

#### Spirometry

All patients will be provided with a hand-held spirometer (*my*SpiroSense; PARI GmbH) to complete regular lung function tests at home (Additional file 3) and to record lung function at the beginning and end of an exacerbation. The *my*SpiroSense spirometer is a digital, self-calibrating instrument. Patients will bring the *my*SpiroSense to study visits so that its data can be imported to computers on site. The spirometry data can be viewed using the SpiroSense*Pro* software and additionally the database can be transformed and exported as a Microsoft Excel (.xls) file and viewed.

#### Exacerbation management

During the treatment period, if patients have symptoms of exacerbation for 48 h or feel that they require antibiotic therapy, they will be asked to call the study team. Exacerbations will be defined as per the recent consensus [31]. A comprehensive exacerbation management plan is detailed in Additional file 2. In general, patients will have rescue medication at home to facilitate management of exacerbations remotely. The trial will use an adjudication panel to categorise exacerbations.

#### Respiratory and Systemic Symptoms Questionnaire (RSSQ)

A member of the research team will administer the RSSQ questionnaire at each study visit to capture changes in the predefined signs and symptoms relative to normal day-to-day fluctuations [32]. It covers a range of patient-reported outcomes relating to cough, sputum, haemoptysis, dyspnoea, lethargy, sinuses, appetite and fever [33]. Modified versions of the RSSQ will be used to capture details of potential exacerbations reported between study visits.

#### Health-related quality of life (HRQoL) questionnaires

Three HRQoL questionnaires will be used: QoL-B, SGRQ and EQ-5D-5 L. The QoL-B assesses symptoms, functioning and HRQoL specific to patients with BE [29, 34, 35]. The SGRQ measures health impairment [2, 34]. The EQ-5D-5 L provides a simple descriptive profile and a single index value for health status [30].

#### Health Service Use Questionnaire

A questionnaire and log will be used to capture participants’ health service use over the study period, including details of medications prescribed (including antibiotics). This will be used for the health economic analysis.

#### Treatment Satisfaction Questionnaire for Medication

At each study visit, participants (except those randomised to the standard care group) will be asked what they think about the effectiveness, side effects and convenience experienced when using the medication over the last 2–3 weeks, or since they last used it. Patients assigned to the group combining HTS and carbocisteine (intervention group 3) will be asked to complete separate questionnaires for each treatment.

#### Schedule of assessments

**Fig. 1:**
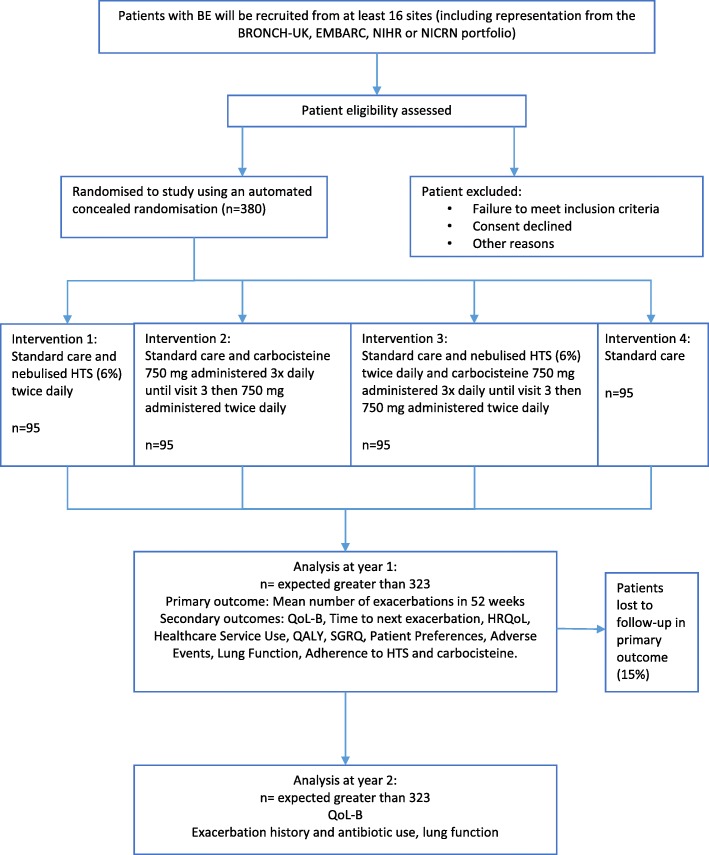
Study schematic. BE, bronchiectasis; BRONCH-UK, UK Bronchiectasis Registry; EMBARC, European Multicentre Bronchiectasis Audit and Research Collaboration; NIHR, National Institute for Health Research; NICRN, Northern Ireland Clinical research Network; HTS, hypertonic saline; QoL-B, Quality of Life - Bronchiectasis; HRQoL, health-related quality of life; QALY, quality-adjusted life year; SGRQ, Saint George’s Respiratory Questionnaire

All patients will be evaluated during the study according to the schedule of assessments outlined in Fig. 1 and Table 1. See also Additional file 8 for the SPIRIT checklist.

**Table 1:**
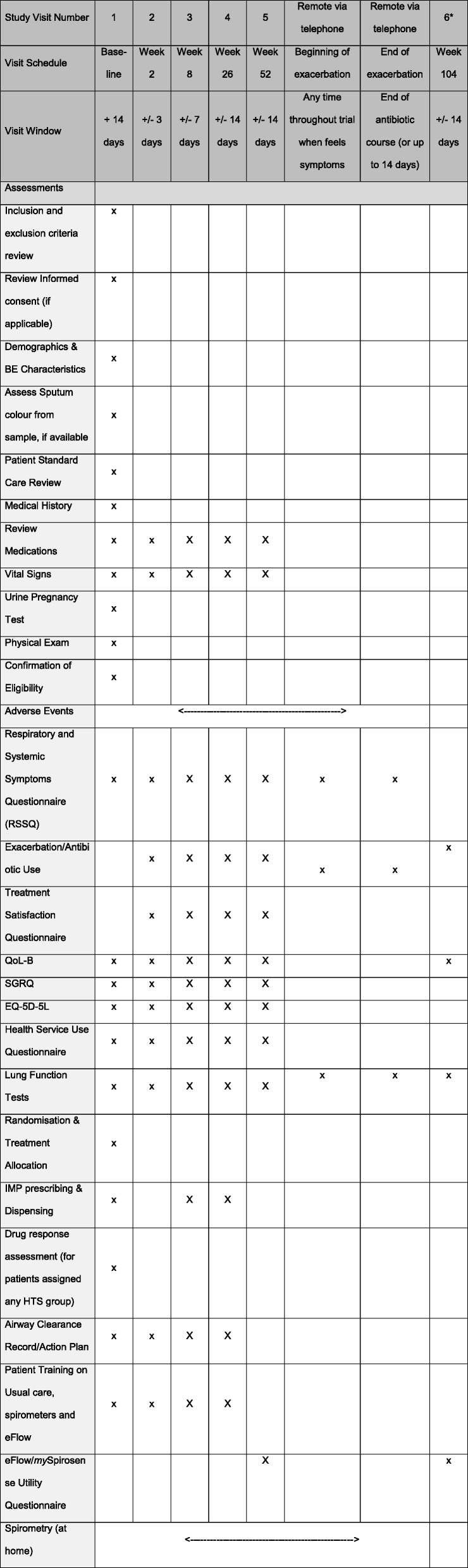
Schedule of assessments

*It is planned that week 104 data will be collected from the European Multicentre Bronchiectasis Audit and Research Collaboration (EMBARC) or UK Bronchiectasis Registry (BRONCH-UK). If this is not possible, the participant will be asked to visit the research site for the data to be collected. BE, bronchiectasis; QoL-B, Quality of Life - Bronchiectasis; SGRQ, Saint George’s Respiratory Questionnaire; EQ-5D-5L, EuroQoL five dimension five level questionnaire

### Sample size

The required sample size is 380 patients including the internal pilot. Based on the primary outcome of mean exacerbations during 52 weeks and a pooled standard deviation of 0.9 exacerbations [36], and assuming the mean number of exacerbations in the control group is 0.7, 216 patients would be sufficient to detect a mean difference in exacerbation rate between groups of 0.4, with 90% power and at the 5% significance level. To allow for a potential interaction between the two interventions, 50% inflation has been included, requiring 324 patients. Further, compensating for 15% dropout gives the total required of 380 patients (95 in each of the four groups). In regard to secondary outcomes, this sample size would provide over 90% power to detect a minimally important difference of 8 points for the QoL-B scale (standard deviation of 18) at the 5% significance level [18, 29] and a 75% increase in median time to exacerbation at 98% power. It would also be sufficient to detect a medium effect size for the other secondary outcomes, at 95% power and 5% level of significance.

### Recruitment

Potential participants may be identified through the EMBARC and BRONCH-UK registries at each of the participating sites, through referrals or while in clinics. Twitter and Facebook accounts (https://twitter.com/TrialCLEAR; https://www.facebook.com/TrialCLEAR/) are being used to encourage engagement and awareness of the trial. The study team will have regular teleconferences with sites to review screening and recruitment figures and resolve any issues.

### Randomisation and blinding

Treatment allocation at each site will be assigned using a concealed automated randomisation process provided by an external organisation. Participants who give their consent will be allocated using a fixed block size to one of the four groups (three intervention groups or one standard care group) in a 1:1:1:1 ratio using a central randomisation system. Randomisation will be stratified by (1) site, (2) exacerbations in the last year (2–3 times, > 3 times) (to minimise baseline imbalances in antibiotic use) and (3) current use of macrolides (yes, no). The trial is open label, and patients, investigators and outcome assessors will be aware of treatment allocation.

### Data collection, quality and procedures

All data collected during study visits and telephone calls with each patient will be recorded in the CLEAR source documents/electronic case report form (CRF). If a participant withdraws during their first year on the study, they will be asked to attend follow-up visits for collection of outcome data. If they do not wish to attend outcome data collection, permission will be sought to access medical notes for collection of data relevant to the trial e.g. the use of antibiotics. If a participant withdraws from all parts of the study, their anonymised data (recorded up to the point of withdrawal) will be included in the study analysis. All patient data will be anonymised.

### Data management

Trial data will be entered onto the electronic case report form (CRF) on a clinical trial database (MACRO) by delegated site personnel and processed electronically as per the Northern Ireland Clinical Trial Unit’s (NICTU) standard operating procedures (SOPs) and the study-specific data management plan (DMP). Data queries will be “raised” electronically using MACRO where clarification from site staff is required for data validations or missing data. Site staff will respond electronically to data queries, ensuring that necessary amendments are made to the clinical trial database.

### Statistical analysis

Baseline characteristics, follow-up measurements and safety data will be described using descriptive summary measures depending on the scale of measurement. The primary analysis will be conducted on a modified intention-to-treat basis consisting of randomised participants with data from at least one post-baseline efficacy assessment. A per-protocol analysis may also be conducted to compare treatment groups. Groups will be compared for the primary outcome (number of exacerbations over 52 weeks) and antibiotic use (number of days of antibiotic use over 52 weeks) using negative binomial regression adjusted for baseline characteristics and other covariates. The QoL-B and other continuous outcomes will be compared between groups using analysis of covariance (ANCOVA) adjusting for baseline characteristics and other covariates. The trial’s 2 × 2 factorial design permits the separate testing of the effects of HTS and carbocisteine on HRQoL and the detection of any interaction between them. These tests will be implemented using three contrasts (representing HTS, carbocisteine and the interaction) in the models. For time to next exacerbation, Kaplan-Meier curves will be prepared and the log-rank test applied to compare the groups. Analyses will be two-sided and tested at an a priori significance level of *p* = 0.05. The primary time point has been defined as 52 weeks after randomisation. There is no adjustment for multiple testing at the different time points, because the primary outcome has been pre-defined and prioritised. Standard approaches will be used to detect missing data.

### Health economics evaluation

A within-trial economic evaluation will assess the cost-effectiveness of the four treatment options at 26 and 52 weeks from the perspective of the NHS and Personal Social Services. A within-the-table analysis will be performed, treating the four groups in the factorial design as mutually exclusive treatments. Economic outcomes will then be estimated and presented separately for each treatment option so that the effect of any interactions can be seen directly. We will estimate the cost per QALY gained, the cost per exacerbation avoided and the net benefit (NB) in each of the treatment groups. Regression analysis with an interaction term will be performed, as a robustness check and to control for baseline covariates. Participants’ health service use and prescriptions (both related and unrelated to their BE) will be prospectively collected from baseline to 52 weeks using logs and questionnaires administered as per Table 1. Costs will be calculated by attaching appropriate unit costs from national sources. QALYs will be calculated using responses on the EQ-5D-5 L over the study period. Uncertainty surrounding the incremental cost-effectiveness ratios will be summarised in cost-effectiveness acceptability curves showing the probability of the therapeutic strategies being cost effective at different threshold levels of willingness to pay per QALY and per exacerbation avoided. Sensitivity will be analysed to explore the impact on cost effectiveness of variations in key parameters. Detailed statistical and health economic analysis plans will be finalised before the commencement of analysis.

### Monitoring arrangements

The trial will be monitored on site in accordance with the trial monitoring plan. This will be an on-going activity from the time of initiation until trial close-out and will comply with the principles of Good Clinical Practice (GCP) and applicable regulatory requirements. The Data Monitoring and Ethics Committee (DMEC) will safeguard the rights, safety and wellbeing of trial participants, monitor data and make recommendations to the Trial Steering Committee (TSC) on whether there are ethical or safety reasons why the trial should not continue. They will monitor the overall conduct of the study to ensure the validity and integrity of the study findings, and will meet annually. The DMEC will comprise independent members with at least one statistician and two respiratory specialists. A DMEC charter will detail the terms of reference of the DMEC, including membership and roles and responsibilities.

### Adverse events

All adverse events (AEs) that are directly observed and spontaneously reported by the patient will be recorded on their CRF. Signs and symptoms of pulmonary exacerbations collected as outcomes of the trial will not be reported as AEs. Therefore, if a patient requires hospitalisation or prolongation of existing hospitalisation as a result of an exacerbation, this will not be reported as a serious adverse event (SAE). The Principal Investigator or designee will assess severity, seriousness, causality, severity and expectedness for each AE and these will be reported in keeping with regulatory requirements.

### End of study

The main trial analysis will be conducted at 52 weeks; however, the formal end of the study will be at the end of the 104-week follow up to establish mucoactive drug use in participants. The trial will be stopped prematurely if mandated by the responsible Research Ethics Committee (REC), Medicine and Healthcare Products Regulatory Agency (MHRA), Sponsor (e.g. following advice from the TSC based on recommendations from the DMEC) or if funding for the trial ceases. The REC that originally gave a favourable opinion of the trial and the MHRA who issued the Clinical Trial Authorisation (CTA) will be notified in writing once the CLEAR trial has been concluded or if it is terminated early.

### Site training

All sites will undertake comprehensive site initiation visits (SIV). PARI or the research team will provide training to sites on the eFlow nebuliser system with eTrack controller, SpiroSense*Pro* and *my*SpiroSense spirometer. Follow-up refresher training will be delivered prior to the first patient’s first visit, and sites will be advised to send questions to the research team at any time. A document containing frequently asked questions will be maintained and circulated to sites, along with a regular newsletter detailing any updates and news on the trial, such as recruitment milestones.

### Trial management arrangements

Trial-specific oversight committees will be convened for the CLEAR trial. These will include a Trial Management Group (TMG), TSC and DMEC. The NICTU will facilitate the setting up and the co-ordination of these committees. All study amendments will be managed by the NICTU and communicated appropriately.

### Patient and public involvement

Service users are involved in the CLEAR trial in both a consultative and collaborative capacity and have influenced this protocol, including the choice of interventions and outcomes to measure. The Chair of Primary Ciliary Dyskinesia Family Support Group UK and a BE carer is a co-applicant on the trial grant and a member of the TSC. The study is registered with the INVOLVE open-access database, which registers research healthcare projects involving members of the public as partners in the research process.

### Data sharing and data access

Requests for data sharing will be reviewed on an individual basis by the Chief Investigator (CI) and TMG. Following the publication of the trial’s main outcomes, there may be scope to conduct additional analyses on the data collected. In such instances, formal requests for data will be made to the CI for discussion with the TMG. If publications might arise from such analyses, those responsible will provide the CI with a copy of the intended manuscript for approval prior to submission to a journal.

## Discussion

CLEAR is a pragmatic effectiveness trial. It was designed as an open-label trial (in preference to a blinded trial) because of factors including the prohibitive cost and feasibility of conducting a trial with blinded patients, practitioners and/or outcome assessors. There is increasing support for trials to use more pragmatic research designs [37], especially in therapy trials where blinding is not feasible [38] or practical and when the funding required to implement blinded trials could be better used to optimise rigour in other areas of trial delivery (Anand R, Norrie J, Bradley JM, McAuley DF, Clarke M: Fool’s gold? Why double blind trials are not always best, submitted). The CLEAR trial also has added value through the embedding of low-cost sub-studies to resolve important uncertainties about trial methods [27].

The findings of the CLEAR trial will be published in a peer-reviewed journal and will help inform national and international guidelines on the use of HTS and carbocisteine as mucoactive drugs for treatment of BE. A lay person’s summary will be sent to local and national patient support and liaison groups, including the European Lung Foundation BE Patient Advisory Group and the British Lung Foundation as well as similar organisations in devolved nations of the UK. A report of the study findings will be provided for the INVOLVE registry. Following journal publication, key findings will be posted on institutional websites that are freely available to the general public and others.

In summary, HTS and carbocisteine are the two most commonly used mucoactive agents in BE, being prescribed to up to 20% of patients but without a solid evidence-base for their effectiveness. The CLEAR trial will demonstrate whether these one-in-five patients with BE are being asked to take burdensome medications that are ineffective or, if effective, whether more consideration needs to be given to prescribing these medications to the remaining 80% of patients with BE.

## Trial status

Recruitment to the CLEAR trial started in July 2018 and at the time of proof reading of this manuscript (December 2019), 130 patients had been recruited, with recruitment aimed to be completed by August 2020. Analysis will begin after recruitment and follow up are completed and the database has been cleaned and locked. The current protocol version is 3.0 (14/05/2018).

## Supplementary information

**Additional file 1.** Validity and sensitivity of the EMBARC definition for exacerbations in bronchiectasis: a sub-study within the CLEAR trial.

**Additional file 2.** Exacerbation management guideline.

**Additional file 3.** Spirometry sub-study.

**Additional file 4.** Optimising recruitment and retention: implementing studies within a trial (SWAT) in the CLEAR trial.

**Additional file 5.** List of Investigative Sites.

**Additional file 6.** Informed Consent Form.

**Additional file 7.** Patient Information Sheet.

**Additional file 8.** SPIRIT 2013 Checklist: Recommended items to address in a clinical trial protocol and related documents*.

## Data Availability

Not applicable.

## Abbreviations

AE: Adverse event
ANCOVA: Analysis of covariance
ANOVA: Analysis of variance
AR: Adverse reaction
BE: Bronchiectasis
BHSCT: Belfast Health and Social Care Trust
BRONCH-UK: UK Bronchiectasis Registry
BTS: British Thoracic Society
CF: Cystic fibrosis
CI: Chief Investigator
CRF: Case report form
CRN: Clinical Research Network
CTA: Clinical Trial Authorisation
CTU: Clinical Trials Unit
DMEC: Data Monitoring and Ethics Committee
DMP: Data management plan
EMBARC: European Multicentre Bronchiectasis Audit and Research Collaboration
EQ-5D-5 L: EuroQoL five dimension five level questionnaire
EudraCT: European Clinical Trials Database
FEF: Forced expiratory flow
FEV_1_: Forced expiratory volume in one second
FVC: Forced vital capacity
GCP: Good Clinical Practice
HRCT: High-resolution computed tomography
HRQoL: Health-related quality of life
HTA: Health and Technology Assessment Programme
HTS: Hypertonic saline
IB: Investigator brochure
ICH: International Conference of Harmonisation
IMP: Investigational medicinal product
IRB: Institutional Review Board
ISRCTN: International Standard Randomised Controlled Trial Number Register
MACRO: Clinical Trials Database
MHRA: Medicine and Healthcare Products Regulatory Agency
NHS: National Health Service
NICTU: Northern Ireland Clinical Trials Unit
NIHR: National Institute for Health Research
NMB: Net monetary benefit
PI: Principal Investigator
PICO: Population, intervention, comparison and outcome(s)
PPI: Patient and public involvement
QALY: Quality-adjusted life year
QoL-B: Quality of Life - Bronchiectasis
REC: Research Ethics Committee
RSI: Reference safety information
RSSQ: Respiratory and Systemic Symptoms Questionnaire
SAE: Serious adverse event
SAP: Statistical analysis plan
SAR: Serious adverse reaction
SGRQ: Saint George’s Respiratory Questionnaire
SOPs: Standard operating procedures
SPC: Summary of product characteristics
SUSAR: Suspected unexpected serious adverse reaction
SWAT: Study within a trial
TMG: Trial Management Group
TSC: Trial Steering Committee
WTP: Willingness to pay
