# Supplementary material for: A 2 × 2 factorial, randomised, open-label trial to determine the clinical and cost-effectiveness of hypertonic saline (HTS 6%) and carbocisteine for airway clearance versus usual care over 52 weeks in adults with bronchiectasis: a protocol for the CLEAR clinical trial
Source: Trials. 2019 Dec 19;20:747. doi: 10.1186/s13063-019-3766-9 (PMC6921594; doi:10.1186/s13063-019-3766-9)
Supplement: Supplementary file 1 — Additional file 1. Validity and sensitivity of the EMBARC definition for exacerbations in bronchiectasis: a sub-study within the CLEAR trial. [file 13063_2019_3766_MOESM1_ESM.docx]

## Additional file 1:

## Validity and sensitivity of the EMBARC definition for exacerbations in bronchiectasis: A sub-study within the CLEAR trial

### Introduction:

There is ambiguity surrounding the definition of bronchiectasis (BE) exacerbations with regards to what signs, symptoms and measurements contribute towards a diagnosis of an exacerbation. BE exacerbations are historically modelled on variations of Fuch’s criteria, which originated in cystic fibrosis (1) and defined an exacerbation as the need for antibiotics following changes in four out of twelve signs or symptoms. Modifications of the Fuch’s criteria are the most commonly used definition for BE exacerbations. They have been used in many prospective and retrospective BE studies to measure exacerbations, depending on what signs or symptoms the investigators think should constitute a BE exacerbation in the context of their research (2-6).

The various combinations of signs and symptoms used to measure exacerbations can lead to differences in the number and duration of these reported across trials in patients with BE. This lack of consensus makes it difficult to compare, contrast and combine the findings for exacerbations across different studies, limiting the use of meta-analyses to resolve important uncertainties about treatment effects.

Recently, a standard consensus definition was released for measuring pulmonary exacerbations specific for BE (Table 1). This followed a 2016 meeting of BE experts from across the world at the first World Bronchiectasis Conference in Germany (7). They developed a consensus definition, which is now referred to as the EMBARC (European Multicentre Bronchiectasis Audit and Research Collaboration) definition.

CLEAR provides an important opportunity for this definition to be explored and compared for the first time against a conventional modified Fuch’s criteria for measuring pulmonary exacerbations.

Table 1: Summary of the three parts of the EMBARC definition for bronchiectasis exacerbations

| **Criteria for the EMBARC definition** | |
| --- | --- |
| The EMBARC definition consists of three parts which must all be satisfied for a fully qualifying exacerbation. | |
| *Part 1- Symptoms* | Patient must present with deterioration in 3 or more of the 6 following symptoms:  1. Cough  2. Sputum volume *and/or* Consistency  3. Sputum purulence  4. Breathlessness *and/or* Exercise tolerance  5. Fatigue *and/or* Malaise  6. Haemoptysis |
| *Part 2- Time* | Symptoms must be present for at least 48 hours. |
| *Part 3- Treatment* | A change in bronchiectasis treatment not limited to antibiotics. |
| For separate exacerbations there must be an unequivocal resolution of symptoms from the first event and >14 days to the commencement of a subsequent event. If this criterion is not met then the exacerbation is counted as a single continuous event. | |

###

### Study Aims:

Specific objectives of this sub-study are:

1. To compare the number of pulmonary exacerbations and treatment changes (prescribed antibiotics) within CLEAR that meet the EMBARC compared to the modified Fuch’s definitions.
2. To explore the signs/symptoms (or combinations) that do not meet either criteria for an exacerbation but still result in a change of treatment.
3. To explore changes in the signs/symptoms from the beginning of an exacerbation to its resolution within both definitions.

### Methods:

#### **Collecting signs and symptoms to diagnose exacerbations**

When a patient suspects an exacerbation, their signs and symptoms will be captured by the CLEAR team over the telephone or at an unscheduled visit, using the Respiratory and Systemic Symptoms Questionnaire (RSSQ) (8). This covers criteria that are required for both the EMBARC and modified Fuch’s definitions and provides an objective way to collect signs and symptoms. It is summarised in Table 2. A key point of the RSSQ is that it recognises the variability of symptoms between and within individuals and so aims to capture acute changes that vary significantly from day-to-day fluctuations.

The RSSQ will be administered with the following modifications:

1. Start of an exacerbation: the item “since the last visit” is modified to capture changes or new symptoms that have already lasted at least 48 hours. This will be referred to as the “symptoms of exacerbation version”.
2. End of exacerbation: “since the last visit” is modified to any changes since the start of a course of antibiotics. This will be referred to as the “end of exacerbation version”.

A question will be added to both versions of the RSSQ to facilitate retrospective scoring on the modified Fuch’s definition to cover wheezing (Question 15).

Insert Table 2 here

#### **Data Collection for Exacerbations:**

Exacerbations will be captured from the patients via the RSSQ and recorded by a member of the CLEAR study team by telephone or during an unscheduled visit. Patients will be advised to wait at least 48 hours from the onset of signs/symptoms before contacting the study team.

When a patient makes contact, the study staff will administer the RSSQ questionnaire (symptoms of exacerbation version) to the patient. In addition to the completion of the RSSQ at the time of an exacerbation, patients will also complete spirometry.

The site staff will use the signs and symptoms information collected from the RSSQ results to assess for an exacerbation. Staff will then discuss these findings and the status of the patient with physicians at the site as to whether a prescription for antibiotics or any other change to the patient’s treatment is required and communicate this to the patient. Any such decision will be ultimately based on the clinical judgment of the responsible clinician.

Insert Table 3 here:

### Calculation of EMBARC and Fuch’s definitions

The answers on the RSSQ (symptoms of exacerbation version) will be assessed using the methodology outlined in Tables 4 and 5 to ascertain whether the signs and symptoms meet the criteria for an EMBARC definition and the modified Fuch’s definition. This will be undertaken by external independent adjudication panels for EMBARC and an internal panel for Fuch’s. Under the Question Number column, if more than one question is listed with ‘**and/or**’ as a separator, then only one of the questions and respective answers is needed to qualify as a deterioration in that domain. If one of the below answers is given and the time is greater than 48 hours, then a deterioration in that domain has occurred.

Insert table 4 here:

An EMBARC exacerbation will be classified as deterioration in at least three of the six domains.

Insert table 5 here:

A modified Fuch’s exacerbation will be classified as deterioration in at least four of the seven domains.

* *Defined as a previously recorded value from a last stable study visit where possible, as these are the main lung functions we will know are done satisfactorily. If a study visit is not considered stable then nearest stable lung function to visit will be considered (9, 10). The percentage decrease will be calculated as:*

$$\frac{FEV1 previously recorded- FEV1 at start of exacerbation}{FEV1 previoulsy recorded} \times100$$

### Statistical Plan:

Descriptive summaries and cross tabulations will be used to compare the proportion of EMBARC-defined exacerbations that meet the criteria against the modified Fuch’s definition and to explore the signs and symptoms that result in a partially qualifying exacerbation. Mean number of exacerbations will be measured from the number of exacerbations per patient over 52 weeks, as defined by each definition. Signs and symptoms that do not meet exacerbation criteria will be summarised. Comparisons will be made between the intervention groups in CLEAR using ANOVA with 95% confidence intervals. Appropriate parametric or non-parametric statistics will be applied to compare mean and median differences in signs and symptoms from the start of an exacerbation to those at the resolution.

References

1. Fuchs HJ, Borowitz DS, Christiansen DH, Morris EM, Nash ML, Ramsey BW, et al. Effect of Aerosolized Recombinant Human DNase on Exacerbations of Respiratory Symptoms and on Pulmonary Function in Patients with Cystic Fibrosis. New Eng J Med. 1994; 331(10):637-42.

2. Tsang KW, Tan KC, Ho PL, Ooi GC, Ho JC, Mak J, et al. Inhaled fluticasone in bronchiectasis: a 12 month study. Thorax. 2005; 60(3):239-43.

3. Tsang KWT, Ho P, Lam W, Ip MSM, Chan K, Ho C, et al. Inhaled Fluticasone Reduces Sputum Inflammatory Indices in Severe Bronchiectasis. American Journal of Respiratory Critical Care Medicine. 1998;158(3):723-7.

4. Mao B, Yang J, Lu H, Xu J. Asthma and bronchiectasis exacerbation. European Respiratory Journal. 2016;47(6):1680-6.

5. Finklea JD, Khan G, Thomas S, Song J, Myers D, Arroliga AC. Predictors of mortality in hospitalized patients with acute exacerbation of bronchiectasis. Respiratory Medicine 2010;104 (6):816-21.

6. O'Donnell AE, Barker AF, Ilowite JS, Fick RB. Treatment of Idiopathic Bronchiectasis With Aerosolized Recombinant Human DNase I. Chest. 1998;113(5):1329-34.

7. Hill AT, Haworth CS, Aliberti S, Barker A, Blasi F, Boersma W, et al. Pulmonary exacerbation in adults with bronchiectasis: a consensus definition for clinical research. European Respiratory Journal. 2017;49 (6):pi 1700051.

8. Lymp J, Hilliard K, Rosenfeld M, Koker P, Hamilton A, Konstan M. Pulmonary Exacerbations in a Phase 2 Clinical Trial of BIIL284Bs in Cf: Development and Implementation of a Respiratory and Systemic Symptoms Questionnaire (RSSQ). Pediatric Pulmonol., 2009; 288.

9. Serisier DJ, Martin ML, McGuckin MA, Lourie R, Chen AC, Brain B, et al. Effect of Long-term, Low-Dose Erythromycin on Pulmonary Exacerbations Among Patients With Non–Cystic Fibrosis Bronchiectasis: The BLESS Randomized Controlled Trial. Journal of the American Medical Association. 2013; 309 (12):1260-7.

10. Barker AF, O'Donnell AE, Flume P, Thompson PJ, Ruzi JD, de Gracia J, et al. Aztreonam for inhalation solution in patients with non-cystic fibrosis bronchiectasis (AIR-BX1 and AIR-BX2): two randomised double-blind, placebo-controlled phase 3 trials. Lancet Respiratory Medicine. 2014, 2 (9):738-49.

Table 2: Summary of the RSSQ questions and answers used in this substudy

| **RSSQ questionnaire summary** | | | | | | | | | | | | | | |
| --- | --- | --- | --- | --- | --- | --- | --- | --- | --- | --- | --- | --- | --- | --- |
| **Questions (answered by patient)** | **Possible Answer Choices** | | | | | | | | | | | | | |
| *1. Increased sputum production* | Much more | A little more | | | | No change | | | | A little less | | Much less | | Never experienced symptom |
| *2.1. Sputum thickness* | Much thicker | A little thicker | | | | No change | | | | A little thinner | | Much thinner | |  |
| *2.2. Sputum Colour* | Worse | | No change | | | | | | | | Better | | |  |
| *3. Increased chest congestion* | Large increase | A little increase | | | No change | | | | | A little decrease | | Large decrease | | Never experienced symptom |
| *4. New or increased coughing up of blood* | Large increase | A little increase | | | No change | | | | | A little decrease | | Large decrease | | Never experienced symptom |
| *5.1. Intensity of cough* | Much harder | A little harder | | | No change | | | | | A little lighter | | Much lighter | | Never experienced symptom |
| *5.2. Frequency of cough* | Much more often | A little more often | | | No change | | | | | A little less often | | Much less often | |  |
| *6. Decreased exercise tolerance* | Much harder | A little harder | | | No change | | | | | A little easier | | Much easier | |  |
| *7.Increased dyspnea with exertion* | Much more difficult | A little more difficult | | | No change | | | | | A little easier | | Much easier | |  |
| *8. Malaise, fatigue or lethargy* | Much more tired | A little more tired | | | No change | | | | | A little more energy | | Much more energy | |  |
| *9.Fever* | Yes | | | | | | No | | | | | | |  |
| *10.Weight loss* | Large weight gain | A little weight gain | | | | No change | | | | A little weight loss | | Large weight loss | |  |
| *11.Sinus pain and tenderness* | Yes | | | | | | | No | | | | | |  |
| *12.Change in sinus discharge* | Worse | | | No change | | | | | | | Better | | | Never experienced symptom |
| *13.School or work absenteeism (due to illness)* | Yes | | | | | | | | | No | | | |  |
| *14.Decreased appetite* | Large increase | A little increase | | | | No change | | | | A little decrease | | Large decrease | |  |
| 1. *Wheezing* *(Additional Question)* | Large increase | A little increase | | | | No change | | | A little decrease | | | | Large decrease | Never experienced symptom |

Table 3: Schedule of assessments to measure exacerbations

| **Visit:** | **In person at site** | **Remote via telephone** | **Remote via telephone** |
| --- | --- | --- | --- |
| Timepoint: | Scheduled study visits within CLEAR | Beginning of exacerbation | End of exacerbation |
| Visit Window: | Visits 2, 3, 4 and 5 | Any time throughout trial when feels symptoms | End of antibiotic course (or up to 14 days) |
| *Review of medications* | X | X | X |
| *RSSQ*  *(since last visit version)* | X |  |  |
| *RSSQ*  *(symptom of exacerbation)* |  | X |  |
| *RSSQ*  *(end of exacerbation)* |  |  | X |
| *Lung function tests (including FEV_1_ spirometry)* | X | X | X |
| *Prescribe antibiotics or change of treatment, if necessary* |  | X | X |
| *Schedule a follow up call* |  | X | X |

Table 4: EMBARC scoring system

| **Domain** | **Question Number**  **(From RSSQ)** | **Answers that qualify as deterioration**  **(From RSSQ)** | **Response that qualify as deterioration present**  **(Yes or No)** | **Is time >48 hours?**  **(Yes or No)** | **Deterioration in domain?**  **(Yes or No)** |
| --- | --- | --- | --- | --- | --- |
| *Cough* | 5.1  *and/or*  5.2 | “much harder” *or* “a little harder” |  |  |  |
|  |  | “much more often” *or* “a little more often” |  |  |  |
| *Sputum Volume and/or consistency* | 1  *and/or*  2.1 | “much more” *or*  “a little more” |  |  |  |
|  |  | “much thicker” *or* “a little thicker” |  |  |  |
| *Sputum Purulence* | 2.2 | “worse” |  |  |  |
| *Breathlessness and/or exercise tolerance* | 7  *and/or*  6 | “much more difficult” *or* “a little more difficult” |  |  |  |
|  |  | “much harder” *or* “a little harder” |  |  |  |
| *Fatigue and/or malaise* | 8 | “much more tired” *or* “a little more tired” |  |  |  |
| *Haemoptysis* | 4 | “large increase” *or* “a little increase” |  |  |  |
|  |  |  |  | *Total deteriorations:* | $\frac{}{6}$ |

Table 5: Modified Fuch’s scoring system

| **Domain** | **Question Number(s)**  **(From RSSQ)** | **Answers that qualify as deterioration**  **(From RSSQ)** | **Is time >48 hours**  **(Yes or No)** | **Deterioration in domain?**  **(Yes or No)** |
| --- | --- | --- | --- | --- |
| *Change in sputum production (consistency, colour, volume, or haemoptysis)* | 2.1  *and/or*  2.2  *and/or*  1  *and/or*  4 | “much thicker” *or* “a little thicker” |  |  |
|  |  | “worse” |  |  |
|  |  | “much more” *or*  “ a little more” |  |  |
|  |  | “large increase” *or* “a little increase” |  |  |
| *Increased dyspnoea (chest congestion or shortness of breath)* | 3  *and/or*  7 | “large increase” *or*  “little increase” |  |  |
|  |  | “much more difficult” *or* “a little more difficult” |  |  |
| *Increased cough* | 5.1  *and/or*  5.2 | “much harder” *or* “a little harder” |  |  |
|  |  | “much more often” *or* “a little more often” |  |  |
| *Fever (>38°c)* | 9 | “yes” |  |  |
| *Increased wheezing* | 15  (Additional Question) | “large increase” or “little increase” |  |  |
| *Decreased exercise tolerance, malaise, fatigue, or lethargy* | 6  *and/or*  8 | “much harder” *or* “a little harder” |  |  |
|  |  | “much more tired” *or* “a little more tired” |  |  |
| *FEV_1_ decreased 10% from a previously recorded value** | *My*SpiroSense Spirometer | “yes” | N/A |  |
|  |  |  | *Total deteriorations:* | $\frac{}{7}$ |
