# Supplementary material for: A 2 × 2 factorial, randomised, open-label trial to determine the clinical and cost-effectiveness of hypertonic saline (HTS 6%) and carbocisteine for airway clearance versus usual care over 52 weeks in adults with bronchiectasis: a protocol for the CLEAR clinical trial
Source: Trials. 2019 Dec 19;20:747. doi: 10.1186/s13063-019-3766-9 (PMC6921594; doi:10.1186/s13063-019-3766-9)
Supplement: Supplementary file 2 — Additional file 2. Exacerbation management guideline. [file 13063_2019_3766_MOESM2_ESM.docx]

**Additional File 2:**

**Exacerbation Management Guideline**

**The following sections describe the process for managing exacerbations should a patient telephone the site with suspected symptoms.**

Patients in CLEAR will be given a Patient Study Card with the contact details of their site so that they can telephone anytime they feel they are experiencing a pulmonary exacerbation. The symptoms of an exacerbation are greater than the day-to-day fluctuations in symptoms that a patient would normally experience and should last for at least 48 hours.

1. **BEGINNING OF AN EXACERBATION:**

When a patient calls the site, the member of study team will:

1. Identify the patient and what treatment group they are in.
2. Complete the **RSSQ questionnaire (symptoms of exacerbation version)** with the patient.
3. Note the date of the start of signs and symptoms of the current exacerbation.
4. Ask the patient if they did a lung function test earlier that day and, if not, instruct them to do so using the *my*SpiroSense spirometer.
5. Ask the patient about any new concomitant medications or airway clearance techniques prescribed and update the concomitant medication form and airway clearance technique log, if required.

After completing the above tasks, the member of the study team will:

1. Use the RSSQ results to assess the exacerbation in the patient’s case report form (CRF) using the table below:


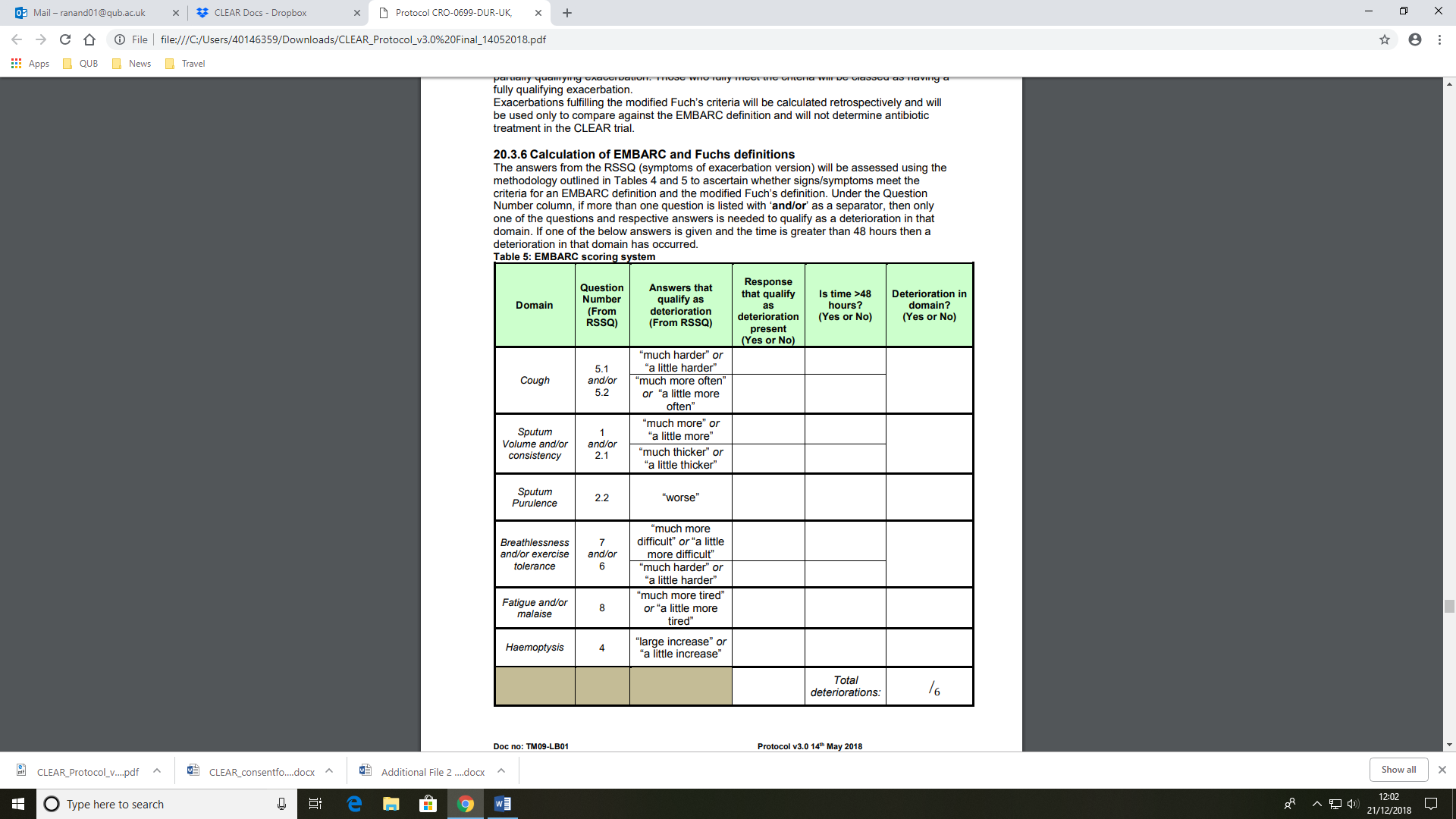


1. Identify if the patient has 3 or more domains deteriorations and if they are present for 48 hours or more.
2. Discuss the patient reported symptoms (and duration of symptoms) with Investigators or other physicians at the site as to whether a prescription for antibiotics or any other change to the patient’s treatment is required and communicate this to the patient.

If the signs and symptoms are inconsistent with an exacerbation, and/or a face-to-face visit is required, the patient should be invited to an unscheduled study visit for further examination.

If the patient has had symptoms for less than 48 hours and antibiotics/change of treatment has not commenced, the study team member should arrange ***a second follow up telephone call for a suspected exacerbation*** and repeat steps 1-8, as above.

If an exacerbation is diagnosed, a member of the study team will do the following:

1. Direct the prescription of antibiotics as instructed by the Investigator (type of antibiotic, dose and length of treatment). This can include the use of rescue antibiotics that patients already have at home. If antibiotics are not prescribed, the reasons for not doing so should be recorded.
2. Record information relating to step 9 alongside any changes in bronchiectasis treatment management in the CRF and concomitant medication form or the airway clearance technique log, if applicable.
3. Schedule a follow-up telephone call with the patient to assess for the resolution of the exacerbation. The date for this will be determined by the end of any antibiotic course prescribed to the patient.
4. **RESOLUTION OF AN EXACERBATION:**

The potential end of the exacerbation is defined as the time when the prescribed antibiotic course is completed. At this time point (or up to 14 days following completion of antibiotic course) the member of the study team will telephone the patient and:

1. Administer the **RSSQ questionnaire (end of exacerbation version).**
2. Direct the patient to undertake a lung function test using the *my*SpiroSense spirometer.
3. Review the concomitant medication form and the airway clearance technique log.

If the exacerbation has not resolved or if another exacerbation begins within 14 days, further antibiotics may be prescribed. If further antibiotics are prescribed within the 14 days, the exacerbation will be counted as one event and the end of this will be when the last antibiotic course is completed.

Flow diagram of exacerbation management:

Symptoms not present for 48 hours then schedule a second telephone call to recheck

(Decision made by PI or delegate)

Patient feels symptoms of potential exacerbation and contacts site to assess for exacerbation

Yes

Follow up by study team for end of exacerbation (which is end of antibiotics course)

Administer RSSQ (end of exacerbation version)

RSSQ (symptoms of exacerbation version) administered a member of study staff and determine do they meet EMBARC definition for exacerbation

Review concomitant medication form and prescribe antibiotics if required

**Exacerbation?**

(Decision made by PI or delegate)

Unable to determine (symptoms are inconsistent or complex)

Unscheduled visit to site for further clinical examination of patient

If exacerbation not resolved or further antibiotics prescribed or then follow up again after antibiotics
