# Supplementary material for: A 2 × 2 factorial, randomised, open-label trial to determine the clinical and cost-effectiveness of hypertonic saline (HTS 6%) and carbocisteine for airway clearance versus usual care over 52 weeks in adults with bronchiectasis: a protocol for the CLEAR clinical trial
Source: Trials. 2019 Dec 19;20:747. doi: 10.1186/s13063-019-3766-9 (PMC6921594; doi:10.1186/s13063-019-3766-9)
Supplement: Supplementary file 3 — Additional file 3. Spirometry sub-study. [file 13063_2019_3766_MOESM3_ESM.docx]

## Additional file 3:

## Spirometry Sub-study

#### **Introduction:**

Measurements of lung function give an indication of the degree of airflow obstruction and disease severity in bronchiectasis (1). Traditionally spirometry is performed when a patient is in clinic under the supervision of a trained healthcare provider and according to standard criteria (2). Technology advances in spirometry equipment have increased opportunities for home monitoring and low-cost portable devices are now available to use outside the clinical setting. Recent studies in various respiratory conditions have found home spirometry monitoring feasible, indifferent to hospital readings and clinically informative (3-6).

PARI has recently released the SpiroSense system which consists of self-calibrating spirometers. The first component of the system is the SpiroSense*Pro* spirometer which is designed for “in clinic” use and lung function measurements are automatically evaluated in accordance with the standards of the American Thoracic Society (ATS) and the European Respiratory Society (ERS) (7). These standards include assessment of:

1. Start-of-test: whether the patient exhaled rapidly and powerfully enough.
2. End-of-test: whether the patient exhaled for long enough.
3. Forced vital capacity (FVC) repeatability: three acceptable measurements must be available in which the difference between the largest and next largest forced vital capacity per second is less than 150ml.
4. Forced expiratory volume in 1 second (FEV_1_) repeatability: three acceptable measurements must be available in which the difference between the largest and next largest capacity per second is less than 150ml.
5. Calibration: indicates whether the automatic calibration of the device was successful.

Predicted values, percent predicted and Z scores are displayed for SpiroSense*Pro* measurements and use the global lung function 2012 equations (8), with the exception of peak expiratory flow (PEF), FEF_25_ and FEF_50_, which use reference values from the European Coal and Steel Community (ECSC/EGKS) in 1993 (9) or the 1987 (10) and 2003 (11) reference value studies by Zapletal.

The second component of the system is the *my*SpiroSense spirometer. It is a lightweight, portable handheld spirometer designed to be used by patients unsupervised and so allows for spirometry to be conducted at home, outside of clinic visits. The key advantage of the *my*SpiroSense is that it is easy to use and does not require a high level of technical ability. The *my*SpiroSense includes a traffic light display system to provide the patient with visual feedback for their latest measurement for a given lung function parameter. For *my*SpiroSense, a lung function parameter is selected and a predicted value for that patient is stored in the device. A marker appears in the traffic light area of the display screen indicating the percentage of expected value reached: > 80% of expected value shows a green light; 60% - 80% of expected value shows an amber light; < 60% of expected value shows a red light.

Expected values can be set from:

- predicted value based on the 2012 lung function equations.
- personal best values.
- custom values that are freely selectable.

For CLEAR the expected value is set as the patients’ personal best of FEV_1_ performed at baseline.

Spirometry data is stored in the *my*SpiroSense device and can be read directly from the device. It also can be imported into the SpiroSense*Pro* software via the USB interface and be read and displayed alongside SpiroSense*Pro* spirometry data, when the device is brought into clinic.

This sub-study will explore the use of *my*SpiroSense for remote spirometry during periods of stability (weekly measurements and measurements on the morning of study visits) and at the start and end of exacerbations in an adult bronchiectasis population.

**Aims:**

This sub-study will aim to:

1. Explore any difference in lung function parameters of home spirometry compared to spirometry completed on the same day in clinic.
2. Explore patient adherence to weekly home spirometry.
3. Explore the quality of home spirometry.
4. Explore changes in spirometry at the start of an exacerbation and on resolution of an exacerbation and if lung function can be used as a predictor for exacerbations.

**Methods:**

This methodology is embedded within the CLEAR trial.

Spirometry will be conducted at the following times:

- Weekly: Throughout the trial all patients will be advised to complete spirometry at home on a weekly basis using the *my*SpiroSens*e*. They will be asked to conduct 3 readings around the same time of day (and where possible post morning airway clearance treatments).
- On the day of study visits: Patients will be asked to complete home spirometry on the morning of scheduled visits using the *my*SpiroSense before coming into clinic for study visits. Patients will then complete clinic spirometry during their study visit using the SpiroSense*Pro* device under the supervision of a clinician.
- At the beginning of an exacerbation: Upon a patient telephone call suspecting an exacerbation, the study staff will instruct patients to complete a home lung function test using the *my*SpiroSense.
- At end of an exacerbation: Upon a patient telephone call at end of an exacerbation, the study staff will instruct patients to complete a home lung function test using the *my*SpiroSense.

The *my*SpiroSense lung function readings are retained in the memory of the device. Patients will bring their *my*SpiroSense to each study visit and the data will be imported to computers on site. The SpiroSense*Pro* software will display the measurements for the patient as ‘thumbnails’ in the display area sorted in descending chronological order. The background colours of the individual thumbnails identify the measurement types: Green thumbnails taken with a *my*SpiroSense, purple thumbnails taken with a SpiroSense*Pro* and blue is a bronchodilation test. Local site staff will send pseudonymised spirometry data using a secure method in line with local and national requirements for data protection and security to the research teams for analysis.

**Analysis plan:**

Spirometry parameters will include FEV_1_, FVC and FEF_25-75%._ The comparison of home and clinic spirometry (*my*SpiroSense versus SpiroSense*Pro* measurements) will be analysed using the Bland–Altman method. This will be a comparison on visit days and the best of three measurements will be used in the statistical analysis. Appropriate descriptive statistics will be used to summarise adherence to weekly home spirometry and level of quality of home spirometry data. Parametric/non-parametric statistics will be applied to compare changes in spirometry at the start and end of exacerbations. A time dependent Cox proportional hazards model will be used to investigate the impact of changes in lung function on the likelihood of an exacerbation.

**Discussion:**

With the rising cost and duration of clinical trials the validation and use of home spirometry will also aid the more pragmatic design of respiratory trials. Less scheduled visits and subsequent assessments will reduce the overall burden for both patients and clinicians. Technological remote or in-home assessments incorporated into clinical trials are likely to make trials more efficient in terms of cost, duration, adherence, recruitment and retention (12, 13). The results from this sub-study will determine whether home spirometry for bronchiectasis patients is feasible. It will explore agreement between supervised spirometry performed in clinic and home spirometry performed independently by patients. In addition, this study will explore changes in home spirometry at the beginning and end of exacerbations.

**References:**

1. Pasteur MC, Bilton D, Hill AT. British Thoracic Society guideline for non-CFbronchiectasis. Thorax. 2010;65(Suppl 1):i58.

2. Moore VC. Spirometry: step by step. breathe. 2012;8(3):232.

3. Russell A, Adamali H, Molyneaux PL, Lukey PT, Marshall RP, Renzoni EA, et al. Daily Home Spirometry: An Effective Tool for Detecting Progression in Idiopathic Pulmonary Fibrosis. American journal of respiratory and critical care medicine. 2016;194(8):989-97.

4. Moor CC, Wapenaar M, Miedema JR, Geelhoed JJM, Chandoesing PP, Wijsenbeek MS. A home monitoring program including real-time wireless home spirometry in idiopathic pulmonary fibrosis: a pilot study on experiences and barriers. Respiratory Research. 2018;19(1):105.

5. Rodriguez-Roisin R, Tetzlaff K, Watz H, Wouters EF, Disse B, Finnigan H, et al. Daily home-based spirometry during withdrawal of inhaled corticosteroid in severe to very severe chronic obstructive pulmonary disease. Int J Chron Obstruct Pulmon Dis. 2016;11:1973-81.

6. Shakkottai A, Nasr SZ. The Impact of Frequent Home Spirometry on Medication Adherence, Health Outcomes and Quality of Life Among Adolescents with Cystic Fibrosis. In: C67. SUPPURATIVE LUNG DISEASES IN CHILDREN. American Thoracic Society; 2017. p. A6136.

7. Miller MR, Hankinson J, Brusasco V, Burgos F, Casaburi R, Coates A, et al. Standardisation of spirometry. Eur Respir J. 2005;26(2):319.

8. Quanjer PH, Stanojevic S, Cole TJ, Baur X, Hall GL, Culver BH, et al. Multi-ethnic reference values for spirometry for the 3-95-yr age range: the global lung function 2012 equations. Eur Respir J. 2012;40(6):1324-43.

9. Laszlo G. European standards for lung function testing: 1993 update. Thorax. 1993;48(9):873-6.

10. Zapletal A, Šamánek M, Paul T. Lung Function in Children and Adolescents. Karger; 1987.

11. Zapletal A, Chalupová J. Forced expiratory parameters in healthy preschool children (3-6 years of age). Pediatr Pulmonol. 2003;35(3):200-7.

12. Hirsch IB, Martinez J, Dorsey ER, Finken G, Fleming A, Gropp C, et al. Incorporating Site-less Clinical Trials Into Drug Development: A Framework for Action. Clin Ther. 2017;39(5):1064-76.

13. Anderson D, Fox J, Elsner N. Transforming the future of clinical development. Deloitte Insights. 2018;1-25.
