# Supplementary material for: A 2 × 2 factorial, randomised, open-label trial to determine the clinical and cost-effectiveness of hypertonic saline (HTS 6%) and carbocisteine for airway clearance versus usual care over 52 weeks in adults with bronchiectasis: a protocol for the CLEAR clinical trial
Source: Trials. 2019 Dec 19;20:747. doi: 10.1186/s13063-019-3766-9 (PMC6921594; doi:10.1186/s13063-019-3766-9)
Supplement: Supplementary file 4 — Additional file 4. Optimising recruitment and retention: implementing studies within a trial (SWAT) in the CLEAR trial. [file 13063_2019_3766_MOESM4_ESM.docx]

## Additional file 4:

## Optimising Recruitment and Retention: Implementing Studies Within A Trial (SWAT) in the CLEAR trial

### Introduction:

Poor recruitment and retention of participants in a clinical trial has implications for the trial’s statistical power, timeline, internal and external validity, as well as the cost of delivering the trial (1, 2). Many trials struggle with recruitment and retention, and reliable evidence on ways to optimise both recruitment and retention is needed.

One approach for testing the effectiveness of different recruitment or retention methods is to embed a methodology study in an ongoing trial, using a SWAT (Study Within A Trial) design (3). The SWAT concept aims to highlight and identify methodology strategies that could improve future clinical research. Dozens of examples of SWAT studies are now available on the Northern Ireland Methodology Hub’s website (4).

### Aims:

The SWAT in CLEAR will explore the effect of methods used to optimise recruitment and retention. The specific objectives are:

- To determine if the nature of the signature and inclusion of a photograph on the invitation letter or introductory material given to potential participants impacts on their recruitment to the trial.
- To determine if giving enrolled participants a thank you note at the end of each study-related visit impacts on their retention in the trial.

### SWAT to be implemented:

The SWAT have registered in the SWAT Repository Store of the Northern Ireland Methodology Hub and full details can be found on the webpage (4). SWAT A is a variation of SWAT 3, whilst SWAT B is registered as SWAT 53 and SWAT C is registered as SWAT 54. SWAT A and B are focused on the recruitment stage of CLEAR, whereas SWAT C is focused on the retention of enrolled patients.

The SWAT will test:

1. Nature of the signature on the invitation letter in the trial recruitment pack:
2. Invitation letter is personally signed, using wet ink, by the local principal investigator (PI).
3. Invitation letter is generically signed and printed electronically as “The CLEAR Trial Team”
4. Inclusion of a generic doctor-patient photograph on the invitation letter:
5. Invitation letter includes a generic doctor-patient photograph
6. Invitation letter does not include a doctor-patient photograph
7. Giving trial participants a thank you note/card after each study visit:
8. Personalised thank you card, including the patient’s name is signed, using wet ink, by the study staff
9. Generic thank you card, not including patient’s name is generically signed and printed electronically as “The CLEAR Trial Team”
10. No thank you card is given to the participant

### Overall Recruitment Strategy for CLEAR:

The recruitment strategy for CLEAR primarily involves a direct approach to potential participants who are regularly attending a clinic in one of the CLEAR sites or have been referred. When a potential participant is identified and approached, they are told about CLEAR and given a recruitment pack containing an invitation letter, patient information sheet and informed consent form.

### SWAT A and B:

#### Outcome Measures:

1. Primary outcome: Proportion of recipients of each of the four invitation letters who join CLEAR.
2. Secondary outcome: Proportion of recruited participants who received each of the four invitation letters who remain enrolled in CLEAR.

#### Design and Implementation:

A 2x2 factorial randomisation will be used for SWAT A and B to allow a simultaneous comparison of the two types of intervention. The four possible combinations of invitation letter are shown below:

| 2x2 design | | **Photograph** | |
| --- | --- | --- | --- |
|  |  | With Photograph | Without photograph |
| **Nature of Signature** | Personal | *personal signature with photo* | *personal signature without photo* |
|  | Generic | *generic signature with photo* | *generic signature without photo* |

SWAT A and B will be implemented for the generic recruitment packs that are handed to potential participants in person at clinics and recruitment packs that are posted to patients.

Equal numbers of the four types of recruitment pack will be prepared for each site, based on recruitment estimates. Each pack will have a unique Pack Identifying Number on the envelope. The order of packs will be randomised using a block size of 8, so that each bundle of 8 contains two of each type of invitation letter. The bundles will be distributed to sites with instructions not to alter the sequence of the packs in the bundles or the order of the bundles.

When giving a recruitment pack to a potential participant in person, site staff will take the topmost pack from the bundle so that they are handed out in the correct sequence. Before the recruitment pack is given to a patient, the Pack Identifying Number will be recorded against their Patient Identification Number on the screening log. If a site uses more than one member of staff to recruit at a time, the packs will be split into two or more piles, with packs being tracked for sequential use from the bundles in each pile. The unique identifying numbers will be used to link individuals who do or do not enrol into the trial with the type of recruitment pack they were given.

When posting a recruitment pack to a patient, sites will put the address of the potential participant on the envelope containing the recruitment pack.

When a site has limited recruitment packs left, they will request further packs. If they run out of the special packs, they will use the standard CLEAR invitation letters until they receive further special packs. The use of standard invitation letters will be recorded on the screening log but data for these patients will not be included in the analysis. Any sites not participating in the SWAT will use the standard invitation letter.

#### Analysis:

The primary analysis will compare the proportion of participants recruited to CLEAR depending on the type of recruitment pack they received. Secondary analyses will examine retention in CLEAR and the extent and duration of the recruited person’s participation.

#### Schematic Diagram:

Potential participants for the CLEAR trial are directly approached in clinics and screened from databases

Patients who express interest in the CLEAR trial are given a recruitment pack that contains one of the four types of invitation letter

The Pack Identifying Number for the information pack given to a patient is recorded against their Patient Identification Number on the screening log

Pairing Pack Identifying and Patient Identification Numbers will reveal which type of letter was given and allow analysis of effects on recruitment

### SWAT C:

#### Outcome Measures:

1. Primary outcome: Proportion of participants in each of the three randomised groups who remain enrolled in the CLEAR trial.
2. Secondary outcomes: Duration of participant involvement in CLEAR in each of the three randomised groups.

#### Design and Implementation:

Enrolled patients will be randomised to be given a generic thank you card (code 01), a personalised thank you card (code 02) or no thank you card (code 03) at the end of each study visit. The relevant numerical code will be recorded on the patient’s case report form for tracking purposes. Participants at sites not participating in the SWAT will be coded as N/A. The message within the cards will thank patients for their time in attending their visit. The study staff leading the visit will not add anything to the message for patients randomised to receive a generic card but, for patients randomised to receive a personalised thank you card, the study staff member leading that visit will handwrite the patient’s name on the card and sign it. If a patient has been randomised to receive either type of thank you card, the relevant card will be inserted into their study file before each scheduled visit. The timing of the visits at which patients will be given cards by the study staff are overleaf:

| Visit Number: | 1 | 2 | 3 | 4 | 5 |
| --- | --- | --- | --- | --- | --- |
| Visit Time: | Base-  Line | Week 2 | Week 8 | Week 26 | Week 52 |

Patients will not be given thank you cards after unscheduled visits. If a patient withdraws from CLEAR, the date of withdrawal will be recorded in the source data.

#### Analysis:

SWAT C will compare retention between the three randomised groups and the durations of a participant’s involvement in CLEAR**.**

#### Schematic Diagram:

Trial participants are enrolled into the CLEAR trial

Trial participants are randomised using their Patient Identification Numbers to one of the three interventions

At the end of each visit the patient is given the assigned thank you card or no thank you card

References

1. Fisher L, Hessler D, Naranjo D, Polonsky W. AASAP: a program to increase recruitment and retention in clinical trials. Patient Education and Counselling, 2012; 86(3):372-7.

2. Treweek S, Pitkethly M, Cook J, Fraser C, Mitchell E, Sullivan F, et al. Strategies to improve recruitment to randomised trials. Cochrane Database Systemic Review, 2018; 2:MR000013.

3. Treweek S, Bevan S, Bower P, Campbell M, Christie J, Clarke M, et al. Trial Forge Guidance 1: what is a Study Within A Trial (SWAT)? Trials. 2018;19 (1):139.

4. Studies Within A Trial (SWAT) [Internet]. [cited 10 MAY 2018]. Available from: <https://www.qub.ac.uk/sites/TheNorthernIrelandNetworkforTrialsMethodologyResearch/SWATSWARInformation/Repositories/SWATStore/>.
