# Supplementary material for: A 2 × 2 factorial, randomised, open-label trial to determine the clinical and cost-effectiveness of hypertonic saline (HTS 6%) and carbocisteine for airway clearance versus usual care over 52 weeks in adults with bronchiectasis: a protocol for the CLEAR clinical trial
Source: Trials. 2019 Dec 19;20:747. doi: 10.1186/s13063-019-3766-9 (PMC6921594; doi:10.1186/s13063-019-3766-9)
Supplement: Supplementary file 5 — Additional file 5. List of Investigative Sites. [file 13063_2019_3766_MOESM5_ESM.docx]

| **Investigative Sites** | |
| --- | --- |
| **Pilot Study** | **Main Study** |
| Dr Martin Kelly Altnagelvin Area Hospital Glenshane Rd Londonderry BT47 6SB | Dr Timothy Gatheral Royal Lancaster Infirmary Ashton Rd Lancaster LA1 4RP |
| Dr John Hurst Royal Free Hospital Pond St Hampstead London NW3 2QG | Dr Anita Sullivan  Queen Elizabeth Hospital Birmingham  Mindelsohn Way  Birmingham B15 2TH |
| Prof Adam Hill Royal Infirmary Edinburgh 51 Little France Cres Edinburgh EH16 4SA | Dr William Flight  John Radcliffe Hospital  Headley Way  Headington  Oxford OX3 9DU |
| Dr Anthony de-Soyza  Freeman Hospital Freeman Rd High Heaton Newcastle upon Tyne NE7 7DN | Dr Alina Ionescu  Royal Gwent Hospital  Cardiff Rd  Newport NP20 2UB |
| Dr Michael Loebinger  Royal Brompton Hospital Sydney St  Chelsea London SW3 6NP | Dr Georgina Russell  St Mary's Hospital  Praed St  Paddington  London W2 1NY |
| Dr Damien Downey Belfast City Hospital 51 Lisburn Rd  Belfast BT9 7AB | Site 16 to be confirmed |
| Prof James Chalmers  Ninewells Hospital and Medical School James Arrott Dr Dundee DD2 1SY |  |
| Dr Muhammad Anwar Princess Alexandra Hospital Hamstel Rd Harlow CM20 1QX |  |
| Dr Mary Carroll Southampton General Hospital Tremona Rd Southampton SO16 6YD |  |
| Dr Rory Convery Craigavon Area Hospital 68 Lurgan Rd Portadown Craigavon BT63 5QQ |  |
