# Supplementary material for: A 2 × 2 factorial, randomised, open-label trial to determine the clinical and cost-effectiveness of hypertonic saline (HTS 6%) and carbocisteine for airway clearance versus usual care over 52 weeks in adults with bronchiectasis: a protocol for the CLEAR clinical trial
Source: Trials. 2019 Dec 19;20:747. doi: 10.1186/s13063-019-3766-9 (PMC6921594; doi:10.1186/s13063-019-3766-9)

#

A study to compare the effect of two medications; hypertonic saline and carbocisteine with routine care in helping to clear sputum in people with bronchiectasis

# The CLEAR Trial

**PATIENT CONSENT FORM**

**Patient’s name: _________________________________**

**Please initial each box**

1. I confirm that I have read and understood the Patient Information Sheet for the above study dated XX/XX/2019 (Version v4.0) and have been given a copy to keep. I have had the opportunity to ask questions and discuss the study. I understand why the research is being done and any foreseeable risks involved.
2. I understand that my participation is voluntary and that I am free to withdraw at any time, without giving any reason, without my medical care or legal rights being affected.
3. I understand that my medical notes and data collected during the study may be looked at by responsible individuals from the hospital, trial co-ordinating centre, sponsor or regulatory authorities, where it is relevant to my taking part in this research. I give permission for these individuals to have access to my records. I agree to information related to this research being retained at the Belfast Health & Social Care Trust.
4. I give permission for information about me to be analysed in strict confidence by responsible people from the study team.
5. I give permission for health checks and study tests to be carried out by nurses/doctors/physiotherapists from the study team.
6. I agree to anonymised information about my lung function and use of the nebuliser being accessed by the trial coordinating centre, researchers from Queens University Belfast and/or the manufacturer of the nebuliser.
7. I give permission for anonymised written information about how I feel about using the spirometer and nebuliser to be sent to the equipment manufacture.
8. I agree to the data I provide being used in an anonymised format in publications and at conferences and understand that I will not be personally identified.
9. I agree to my GP being informed of my participation in the study and for information about me to be provided to the research team.
10. I give permission for information collected about me by the Bronch-UK or EMBARC registry research studies to be accessed by responsible people from the study team, to facilitate the collection of follow-up data in year 2 of the study.

**I agree to take part in the above study**

**_____________________________ ____________________ __­­­­­­­­­­­­­­­­___________**

**Name of Patient Signature Date**

**(Block capitals)**

**_____________________________ ____________________ __­­­­­­­­­­­­­­­­__________**

**Name of Person taking consent Signature Date**

**(Block capitals)**

1 copy for patient

1 copy for hospital notes

1 original for study site file
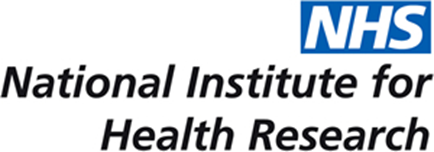

Supplement: Supplementary file 6 — Additional file 6. Informed Consent Form. [file 13063_2019_3766_MOESM6_ESM.docx]
