# Supplementary material for: A 2 × 2 factorial, randomised, open-label trial to determine the clinical and cost-effectiveness of hypertonic saline (HTS 6%) and carbocisteine for airway clearance versus usual care over 52 weeks in adults with bronchiectasis: a protocol for the CLEAR clinical trial
Source: Trials. 2019 Dec 19;20:747. doi: 10.1186/s13063-019-3766-9 (PMC6921594; doi:10.1186/s13063-019-3766-9)
Supplement: Supplementary file 7 — Additional file 7. Patient Information Sheet. [file 13063_2019_3766_MOESM7_ESM.docx]

A study to compare the effect of two medications; hypertonic saline and carbocisteine with routine care in helping to clear sputum in people with bronchiectasis

The **CLEAR** Trial

**PATIENT INFORMATION SHEET**

**We would like to invite you to take part in our research study**.

- Before you decide we would like you to understand why the research is being done and what it would involve for you.
- A study doctor or research nurse will go through this information sheet with you. Please ask questions about anything that might not be clear.
- Please take time to read the information carefully and discuss it with family and friends if you wish.
- Take your time to make your decision about whether or not to take part.

*Thank you for reading this*

| Page | Contents |
| --- | --- |
| 2 | Information about why the study is being done and why you have been invited to take part |
| 3-7 | Detailed information about what would happen if you decided to take part |
| 8-9 | Study Calendar |
| 9-10 | The pros and cons of taking part and what to do if things go wrong |
| 10-11 | Detailed information regarding confidentiality and study results |
| 12 | Important telephone numbers if you have questions about the study or are taking part and feel unwell at home |
| 13-14 | Data Transparency statement letting you know how we will use the information we collect about you |

**Why is this study being done?**

This study is being done to try to find a suitable and effective treatment for the side effects associated with bronchiectasis. Patients with bronchiectasis typically suffer from a persistent cough with chronic daily sputum production. Patients with bronchiectasis can also suffer flare-ups when their bronchiectasis worsens or they develop infections and need additional medicines to treat their symptoms. These flare ups are known as exacerbations. These can affect the quality of life of people living with bronchiectasis.

This study is particularly concerned with exploring which or what combinations of medicines (hypertonic saline and carbocisteine) might help patients bring up their sputum more easily. These medicines are already used sometimes but there is not enough evidence on how effective they are for them to be recommended as part of a patient’s usual care.

Hypertonic saline is salty water that is delivered into the airways via a nebuliser, which converts it into a mist. This treatment usually takes about 5 minutes. Carbocisteine is taken in capsule form. Research has shown that these agents can make it easier for patients to cough up sputum. This can result in fewer exacerbations and hospital admissions, which could improve overall quality of life for people with bronchiectasis.

We want to find out if patients with bronchiectasis have fewer exacerbations if they use hypertonic saline, carbocisteine, or both of these medicines together for 12 months. To get reliable results we need to make a fair comparison so we will also compare these medicines with the usual treatments that patients are given for bronchiectasis at present, this is usually a form of manual airway clearance technique. This research has the potential to impact future care for people with bronchiectasis.

As part of this trial we are completing additional research to look at the way your exacerbations are diagnosed, and this is being looked at as part of a student’s PhD project.

**Why have I been invited to take part?**

We are asking you to consider taking part in this study because you have been diagnosed with bronchiectasis.

**Do I have to take part?**

No. It is up to you to decide whether or not to take part. If you decide to take part, you will be given this information sheet to keep and asked to sign a consent form. By signing the consent form you will be agreeing to take part in the study, for your GP to be informed of your participation in the study and that members of the study team can access your medical notes. You may still decide to withdraw at any time without giving a reason. If you decide not to take part, or if you withdraw, this will not affect your legal rights or healthcare at any time, now or in the future.

What are the alternatives for treatment?

If you decide not to enter this study you will receive the standard treatment for bronchiectasis e.g. bronchodilators, airway clearance techniques. It is your decision whether you would like to take part or not. Please talk to your doctor about the benefits and risks of this.

**What will happen if I take part in this research study?**

Patients who are eligible and agree to take part will be enrolled in the trial for 2 years. 1 year on the study treatment and a follow-up at the end of year 2. You will be asked to visit the clinic 5 times over the year you are on the study treatment. A summary of the visits is shown in the study calendar table below.

**First Visit**

We will arrange a convenient time for you to visit the hospital for Day 1 of the study. You will be given the opportunity to ask questions regarding the study and if you are happy to take part you will be asked to sign a consent form at the end of this document. The study personnel will then:

- Ask you about your medical history and what medications you take or have taken in the last three months.
- Ask you about your bronchiectasis including if you have had to visit the doctor or hospital or take any antibiotics in the last year for exacerbations. Your medical notes may be reviewed to check this and to find out the treatment you received in the past and treatment you currently receive.

You will be asked to produce a sputum sample and the colour of the sputum will be assessed.

- Complete a physical examination. We will also measure your height, weight and vital signs including temperature and blood pressure. We will also ask about your smoking status.
- Ask you to complete questionnaires about how your health affects your quality of life.
- You will be asked about your use of the health service e.g. visits to the GP and hospital

*Lung Function Tests*

You will also be asked to perform some lung function tests. You will be asked to perform spirometry. This is the same as the usual blowing test you receive at clinic (i.e. ‘blow as hard and as fast as you can, all the way out into this tube’). You will need to complete this 3 times for every lung function test.

You will also be provided with a spirometer to allow you to perform lung function tests at home and when you are feeling unwell. You should complete your lung function test when you are unwell as you may be experiencing the onset of an exacerbation and also at the end of an exacerbation. This will provide the study team with important information about your lung function and any changes that may occur during an infection.

We will ask you complete the lung function tests at home every week during the first year of the study. These tests should be completed on the same day and about the same time each week. On the weeks that you have visits to the study site we would like you to complete your spirometry on the morning of your visit. As part of the PhD researcher’s project they will look at how the spirometer you use at home works compared to the spirometer the doctor uses in the clinic.

You will be asked to bring the spirometer with you to every visit to the study site so that we can collect the data about your lung function from the spirometer. The local research team will attach the spirometer to a laptop computer with special software installed that can read and download the data on the spirometer. All of the data will be anonymised and will not have information that can identify you. Your data will be seen by staff involved in the study (staff at the study site and the trial coordinating centre and researchers at Queens University Belfast) and by the company that makes the spirometer when they collect the laptops from the study sites at the end of the study. Any transfer of your data will be done in line with local and national requirements for data protection and security.

*Study Treatment*

Patients who are eligible will be assigned to one of four treatment groups using a technique called randomisation. This means that there is an equal chance of you being allocated to one of four groups. The treatment groups are:

1. **Nebulised hypertonic saline 2 times a day alongside your usual care over 52 weeks**
2. **Treatment with carbocisteine (750 mg three times a day until your 3^rd^ visit then reducing to 750 mg twice a day) over 52 weeks alongside your usual care**
3. **Treatment with both hypertonic saline solution (twice a day) and carbocisteine (750 mg three times a day until your 3^rd^ visit then reducing to twice a day) over 52 weeks alongside your usual care**
4. **Usual care only over 52 weeks**

A computer program will select which group you will be in and you will be told the result. Neither you nor your doctor can choose your group. This study will enrol 380 patients from a number of sites so 95 patients will be allocated to each of the four treatment groups.

If you are allocated to a group treated with carbocisteine you will take 750 mg three times a day until you attend your 3^rd^ visit at the clinic. The study doctor will then change your prescription so that you will take 750 mg of carbocisteine two times a day for the remainder of the 52 weeks treatment period.

If you are allocated to a group treated with hypertonic saline, before you go home with your hypertonic saline you will have an assessment called a ‘challenge test’ completed before the treatment is started. You will be given a test dose to check if you experience any chest tightness and may be given additional therapy (salbutamol) to help relieve this. It is very rare that anyone would have a very bad reaction, but if you feel unwell after it, you may be asked to repeat the test again during this visit or may be asked back to the study site on a different day to repeat the assessment if the doctor thinks this is best. You will only begin your study treatment when the study team is happy with the results of this test. The study doctor will also assess if you should be given salbutamol to take during the study period if you are not taking this already.

If you are allocated to a group treated with hypertonic saline, you will be provided with a nebuliser that has a controller unit that has technology that can collect information on how you are using your nebuliser i.e. when and for how long you are using it. You will need to bring your nebuliser and the controller with you to each of your study visits. During the visit, the research team at the site will link the nebulizer and controller using IT systems with piece of equipment known as a ‘hub’. When this is done the information collected from the nebuliser will be transmitted through IT networks with a high level of security to a secure electronic database held by the company that makes the nebuliser. The research team (your local study team and researchers at the trial coordinating centre and Queens University Belfast) will be given access to the information. Certain personnel at the company that makes the nebuliser and IT personnel that work on their behalf may also access your information if there are any technical problems. All of the data collected will be anonymised and will not include any of your personal details. At different times during the study the information will be sent to the trial-coordinating centre so that they can analyse the results.

This information will not be analysed until your participation in the trial in complete so you and the research team will not know how you are using the nebuliser until the end of the study treatment period at week 52. The study team will review the information with you when you come to the research centre for visit 5. This data is being collected to help us understand the results more fully and help us understand better how patients use nebulisers.

The nebuliser and spirometer provided are only to be used by you for the research study, and should not be given to anyone else. You are not permitted take apart or modify the equipment in any way so if you have any problems with using them you should contact your study team. You will return both the nebuliser and spirometer to the research team at the end of the study.

Depending on the group you are assigned to, you will be given a supply of medicines to take home. You will also be provided with a log to record information about any other medications you take and your use of the health service. When you attend the clinic for visits, 2-5 you will use this log to complete questionnaires about your use of the health service e.g. visits to your GP or Hospital.

You will be given a card with the contact details of your local research team in case you have any problems or if you become unwell. You will also be given a thermometer to record your temperature at home if you are feeling unwell.

If you have gradually worsening symptoms for 2 days or more related to a flare up in your bronchiectasis you should contact the research team during working hours on the number provided on the study card. The research team will ask you questions about your signs or symptoms. They will then arrange any treatment you may need or may invite you to come to the research site to if they need to collect more information or assess you in person. If your symptoms have worsened for 2 days during the weekend or bank holiday you should proceed with taking any rescue antibiotics you have at home or that have been prescribed by your GP and then call the study team as early as possible.

If you feel acutely unwell and feel you need advice or treatment immediately please contact the research team on the number provided which will also be noted on your study card.

You will also be given blank copies of several questionnaires about how your health affects your quality of life to take home to complete at home on the morning of your next study visit.

**Visits 2, 3, 4 and 5.**

You will be asked to attend the hospital for four further visits in year 1:

- 2^nd^ visit- 2 weeks after your first visit
- 3^rd^ visit- 8 weeks after your first visit
- 4^th^ visit- 26 weeks after your first visit
- 5^th^ visit- 52 weeks after your first visit

Just before you come into the clinic for your study visit, we would like you to complete the questionnaires you were given at your last study visit. These should be completed in the specific order instructed by your study team. We do not want you to complete these in front of the study team as we do not want them to influence your responses in any way. You will need to bring the completed copies to your study visit where the study team will check that all required questions have been answered.

At each study visit study personnel will:

- Measure your vital signs e.g. weight, blood pressure
- Ask you about any new or changes to medications you have taken since your last visit
- Check if you have had any possible side effects/complications from your study treatment
- Check if you have had exacerbations or any signs/symptoms of infection and if you have taken any antibiotics
- Check that you have completed the questionnaires about how your health affects your quality of life
- Ask you to complete a questionnaire about your health service use e.g. visits to your GP or hospital using the log you have completed at home
- Ask you to perform lung function tests

Depending on the group you are assigned to, at visit 2, 3, 4 and 5 you will also be asked to complete a short questionnaire on how happy you are with the study treatment**.**

In addition, at visit 3 and 4 you will be provided with more study medication. If you are allocated to a group taking carbocisteine, at visit 3 your study doctor will change your prescription from 750 mg three times a day to 750 mg two times a day.

It is very important for you to return for every study visit. You need to bring your supply of study medication including any empty packs when you come back at visit 3, 4 and 5. Do not throw out the study medication and do not discard the empty boxes.

Study medication will finish at 52 weeks, if you wish to continue treatment with hypertonic saline or carbocisteine after this time you will need to discuss it with your respiratory consultant or your GP. It is up to them to decide if it would be beneficial to prescribe you either of these treatments.

At visit 5, you will also be asked to complete a questionnaire about using the spirometer and nebuliser. The questionnaires will not collect any of your personal details and data will be anonymised. The questionnaires will be sent by your local research team to the trial coordinating centre who will send onto the company that makes the equipment who may use the information for different reasons.

**Year 2 (104 week) follow-up**

When you have been enrolled in the study for 2 years we will collect some additional data. If you are also taking part in either the EMBARC or Bronch-UK research studies, we will ask your permission in the consent form for this study to access your EMBARC or Bronch-UK information. We would use information that would be being collected as part of your yearly follow-up data collected for the EMBARC and Bronch-UK research studies so that we do not have to collect the same data twice We want to record data on whether you have had any exacerbations and have needed any antibiotics, responses to a questionnaire about your health and lung function measurements.

If you are not taking part in the EMBARC or Bronch-UK research studies or you do not want us to access data being collected on these studies, then we will invite you to visit the hospital. At this study visit the research personnel will:

- Check if you have had any exacerbations and signs or symptoms of infection and if you have taken any antibiotics in the past year
- Ask you to complete a questionnaire about how your health affects your quality of life
- Ask you to perform lung function tests

**Study Calendar**

| **Visit** | **What will happen?** |
| --- | --- |
| **1^st^ visit**  **Day 1** | - Review Medical history (including information about your Bronchiectasis) and medications - Complete a physical examination and vital signs e.g. height, weight, blood pressure - Assess sputum colour by sample, or patient will assess using a colour chart - Complete questionnaires about your health and how it affects your quality of life - Complete questionnaire about your use of the health service - Lung function tests in the clinic - Challenge test (depending on which treatment you are receiving) - Provide study medication (if applicable) and any equipment you need |
| **2^nd^ visit**  **Week 2** | - At home, complete a lung function test and the questionnaires about your health and how it affects your quality of life - Vital signs e.g. weight, blood pressure - Review medications - Review any adverse events including any bronchiectasis exacerbations - Complete questionnaires about how happy you are with your treatment - Complete questionnaire about your use of the health service - Lung function tests in the clinic |
| **3^rd^ visit**  **Week 8** | - At home, complete a lung function test and the questionnaires about your health and how it affects your quality of life - Vital signs e.g. weight, blood pressure - Review medications - Review any adverse events including any bronchiectasis exacerbations - Complete questionnaires about how happy you are with your treatment - Complete questionnaire about your use of the health service using the log you completed at home - Lung function tests in the clinic - Collect any empty packs and unused medication and provide study medication (if applicable) |
| **4^th^ visit**  **Week 26** | - At home, complete a lung function test and the questionnaires about your health and how it affects your quality of life - Vital signs e.g. weight, blood pressure - Review medications - Review any adverse events including any bronchiectasis exacerbations - Complete questionnaires about how happy you are with your treatment - Complete questionnaire about your use of the health service using the log you completed at home - Lung function tests in the clinic - Collect any empty packs and unused medication and provide study medication (if applicable) |
| **5^th^ visit**  **Week 52** | - At home, complete a lung function test and the questionnaires about your health and how it affects your quality of life - Vital signs e.g. weight, blood pressure - Review medications - Review any adverse events including any bronchiectasis exacerbations - Complete questionnaires about how happy you are with your treatment - Complete questionnaire about your use of the health service using the log you completed at home - Lung function tests in the clinic - Collect any empty packs and unused medication - Return any study equipment (if applicable) |
| **6^th^ visit**  **Week 104** | - Review any bronchiectasis exacerbations and antibiotic use - Complete questionnaire about your health and how it affects your quality of life - Lung function tests - Return any study equipment (if applicable) |
| **If you have an exacerbation you should:** | - Call your study team on the contact number provided - Describe your symptoms - Complete lung function tests - Visit the research site if requested by the study team |
| **Weekly at home** | - Perform spirometry |

**What are the possible disadvantages and risks of taking part?**

Possible disadvantages of taking part are the inconvenience it may cause you attending the hospital for the additional five visits. Also depending on the group you are allocated to, you may find the new treatment inconvenient as nebulisers are taken twice daily and each treatment takes approximately 5 minutes.

During the study lung function tests will be performed. Discomforts associated with lung function tests may include dizziness, headache or coughing but these occur infrequently.

In a small number of people nebulised hypertonic saline may cause chest tightness. If you are allocated to a group taking hypertonic saline you will have a test called a ‘challenge test’ completed before the treatment is started. You will be given a test dose to check if you experience any chest tightness and may be given additional therapy (salbutamol) which can be taken through the nebuliser to help relieve this. If you experience any chest tightness during the study you will be asked to contact your study team.

Like all medicines, carbocisteine can cause side effects, although not everybody gets them. A small number of people have reported side effects including anaphylactic reactions, vomiting, skin rashes and allergies. There have also been a small number of reports of gastrointestinal bleeding occurring during treatment with carbocisteine so caution needs to be taken if any other medications being taken are known to cause gastrointestinal bleeding. Your study doctor will check your medications at the first visit. You should not take any new medications including prescriptions or over the counter medications unless the study doctor or your GP tells you otherwise. If you are prescribed new medications between your visits, it is very important that you inform the study team at your next visit.

Your study doctor will let you know as soon as any information becomes available that may influence your treatment or your choice to continue participating in the study. If you do not feel well or develop a problem that requires medical attention, please call your study doctor as soon as possible. If any of the side effects become intolerable, the doctor may decide to stop the study medication and you will be asked if you are still willing to be involved with the remainder of the study and continue with the clinic visits.

**What are the possible benefits of taking part?**

The overall aim of the study is to provide information on whether treatment with carbocisteine or hypertonic saline solution reduces the number of exacerbations experienced by patients with bronchiectasis. We cannot guarantee any direct benefits over and above your usual care.

If you are in the group receiving usual care you will be receiving the same treatment most patients receive when they have bronchiectasis.

We hope that the information that we get from the study will help us to treat people with bronchiectasis in the future by providing a greater understanding of which or what combination of medications works best to reduce the number of exacerbations.

**What if something goes wrong?**

The NHS hospital remains responsible for your care during the study. If you have a concern about any aspect of the way you have been approached or treated during the course of this study, please speak to the study doctor or member of the research team for more information. The normal complaints procedure of your local trust is also available to you. More information will be available usually through the Patient Advice and Liaison Service (also known as PALS) or your Trust’s complaints department.

In the event of suffering some harm or injury as a result of taking part in this study, due to someone’s negligence, then you may have grounds for a legal action for compensation but you may have to pay your legal costs.

**What are the costs and payments for taking part in this study?**

You are not responsible for any costs associated with or for attending study visits, examinations, and procedures. You will receive compensation to cover the costs for your participation in this study e.g. transport, parking, and petrol. These funds will be paid at each study visit.

Your study doctor does not receive any personal financial compensation for this study. All money paid to the study doctor goes to pay the cost of equipment, tests and staff to run this study.

**Will information from the study be kept confidential?**

Any information collected about you during the course of the study will be kept strictly confidential and will only be seen by staff involved in the study (which may include hospital staff, staff from the Clinical Trials Unit and researchers at Queens University Belfast) and people from regulatory authorities who ensure that studies such as this are carried out correctly. In addition, certain personnel at the company that makes the nebuliser and IT personnel that work on their behalf will have access to anonymised data about your use of the nebuliser. All of which have a duty of confidentiality to you as a research participant.

Your identity will be anonymised and your name will not be published. You have the right to see your personal health information related to the research study, but you will not be able to review some parts of the information until after the study has finished. When any information from the study is published it will contain no personal information and it will not be possible to identify any individual.

The data from this study will be kept for at least five years after its conclusion and may be used in other research studies. If it is used in this way all personal identifiers will be removed and it will not be possible to identify any individual.

**What will happen to the results of the research study?**

The study will take a number of years to complete. Following this, we hope to present the results at national and international conferences with clinicians and people working with the bronchiectasis population so as the implications of these treatments quickly reach staff involved in managing patients with bronchiectasis. The results will also be published in scientific journals. You can request a copy of the study results from your study doctor.

**Who is organising and funding the research?**

The study is being sponsored by the Belfast Health and Social Care Trust and funded by the National Institute for Health Research Health Technology Assessment Programme. The study is organised by local researchers (contact details below).

**Who has reviewed the study?**

To protect your interests, all research in the NHS is looked at by an independent group of people called a research ethics committee. Ethical approval for this study has been obtained from a national ethics committee. Your local NHS trust has given approval for the study to take place at your hospital/clinic.

**What happens if I have any questions, concerns or complaints about the study?**

If you have any questions about participation in this study or concerns about the way it has been carried out, you should contact your hospital’s Principal Investigator (contact details below).

What will happen if I don’t want to carry on with the study?

You can withdraw from the study at any time without giving a reason and your care will not be affected. You would continue to be looked after by your normal team of hospital doctors and nurses and receive the treatment they felt was best for your infection. If you withdraw from the study, we would use the data collected up to your withdrawal.

**What happens after the study is finished?**

After the study is finished your follow up and on-going care will be through the bronchiectasis clinic as before.

**Who to contact for further information:**

**If you are taking part in the study or feel unwell at home please use the contact details provided on your study card.**

For specific questions about the study you may contact your research doctor or nurse during work hours using the contact details below:

Study Coordinator: ________________________

Telephone number: _________________________

Study Doctor: ________________________

Telephone number: _________________________

Study Research nurse: _________________________

Telephone number: _________________________

**Thank you for reading this information sheet and considering taking part in this study.**


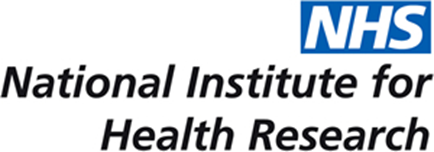


**A study to compare the effect of two medications; hypertonic saline and carbocisteine with routine care in helping to clear sputum in people with bronchiectasis - The CLEAR Trial**

**Data Transparency Statement**

Belfast Health & Social Care Trust is the sponsor for this study based in the United Kingdom. We will be using information from you and/or your medical records in order to undertake this study and will act as the data controller for this study. This means that we are responsible for looking after your information and using it properly. Belfast Health & Social Care Trust will keep identifiable information about you for 5 years after the study has finished.

Your rights to access, change or move your information are limited, as we need to manage your information in specific ways in order for the research to be reliable and accurate. If you withdraw from the study, we will keep the information about you that we have already obtained. To safeguard your rights, we will use the minimum personally-identifiable information possible.

You can find out more about how we use your information at:

[www.belfasttrust.hscni.net/about/DataProtection.htm](http://www.belfasttrust.hscni.net/about/DataProtection.htm)

[Site Name] will collect information from you and/or your medical records for this research study in accordance with our instructions.

[Site Name] will use your name, NHS/Hospital number and contact details to contact you about the research study, and make sure that relevant information about the study is recorded for your care, and to oversee the quality of the study. Individuals from Belfast Health & Social Care Trust and regulatory organisations may look at your medical and research records to check the accuracy of the research study. [NHS site] will pass these details to Belfast Health & Social Care Trust along with the information collected from you and/or your medical records. The only people in Belfast Health & Social Care Trust who will have access to information that identifies you will be people who need to audit the data collection process. The people who analyse the information will not be able to identify you and will not be able to find out your name, NHS/Hospital number or contact details.

[Site Name] will keep identifiable information about you from this study for 15 years after the study has finished.

When you agree to take part in a research study, the information about your health and care may be provided to researchers running other research studies in this organisation and in other organisations. These organisations may be universities, NHS organisations or companies involved in health and care research in this country or abroad. Your information will only be used by organisations and researchers to conduct research in accordance with the UK Policy Framework for Health and Social Care Research.

This information will not identify you and will not be combined with other information in a way that could identify you. The information will only be used for the purpose of health and care research, and cannot be used to contact you or to affect your care. It will not be used to make decisions about future services available to you, such as insurance.
